# Comparative Transcriptomics Analyses across Species, Organs, and Developmental Stages Reveal Functionally Constrained lncRNAs

**DOI:** 10.1093/molbev/msz212

**Published:** 2019-09-20

**Authors:** Fabrice Darbellay, Anamaria Necsulea

**Affiliations:** 1 School of Life Sciences, École Polytechnique Fédérale de Lausanne (EPFL), Lausanne, Switzerland; 2 Laboratoire de Biométrie et Biologie Évolutive, CNRS UMR 5558, Université de Lyon, Université Lyon 1, Villeurbanne, France

**Keywords:** long noncoding RNAs, evolution, development, comparative transcriptomics

## Abstract

The functionality of long noncoding RNAs (lncRNAs) is disputed. In general, lncRNAs are under weak selective pressures, suggesting that the majority of lncRNAs may be nonfunctional. However, although some surveys showed negligible phenotypic effects upon lncRNA perturbation, key biological roles were demonstrated for individual lncRNAs. Most lncRNAs with proven functions were implicated in gene expression regulation, in pathways related to cellular pluripotency, differentiation, and organ morphogenesis, suggesting that functional lncRNAs may be more abundant in embryonic development, rather than in adult organs. To test this hypothesis, we perform a multidimensional comparative transcriptomics analysis, across five developmental time points (two embryonic stages, newborn, adult, and aged individuals), four organs (brain, kidney, liver, and testes), and three species (mouse, rat, and chicken). We find that, overwhelmingly, lncRNAs are preferentially expressed in adult and aged testes, consistent with the presence of permissive transcription during spermatogenesis. LncRNAs are often differentially expressed among developmental stages and are less abundant in embryos and newborns compared with adult individuals, in agreement with a requirement for tighter expression control and less tolerance for noisy transcription early in development. For differentially expressed lncRNAs, we find that the patterns of expression variation among developmental stages are generally conserved between mouse and rat. Moreover, lncRNAs expressed above noise levels in somatic organs and during development show higher evolutionary conservation, in particular, at their promoter regions. Thus, we show that functionally constrained lncRNA loci are enriched in developing organs, and we suggest that many of these loci may function in an RNA-independent manner.

## Introduction

Long noncoding RNAs (lncRNAs, loosely defined as transcripts without protein-coding potential, at least 200 nucleotides long) are an excellent illustration of the ongoing conceptual tug-of-war between biochemical activity and biological function (Graur et al. 2013; Doolittle 2018). Recent sequencing-based studies identified thousands of lncRNAs in vertebrates (Guttman et al. 2009; Khalil et al. 2009; Iyer et al. 2015; Pertea et al. 2018). Although this class of transcripts includes lncRNAs with undisputed biological roles, such as *Xist* (Brown et al. 1991) or *H19* (Brannan et al. 1990), experimental validations are lacking for the great majority of lncRNAs and their functionality is controversial.

The first functional characterizations of individual lncRNAs forged the idea that they are important contributors to gene expression regulatory networks. This has been unequivocally proven for some lncRNAs, such as *Xist*, whose transcription and subsequent coating of the X chromosome triggers a complex chain of molecular events leading to X inactivation in placental mammals (Munschauer et al. 2018). Other proposed mechanisms for gene expression regulation by lncRNAs include directing chromatin-modifying complexes at specific genomic locations, to control gene expression in *trans* (Rinn et al. 2007); providing decoy targets for microRNAs (Cesana et al. 2011); enhancing neighboring gene expression through an RNA-dependent mechanism (Ørom et al. 2010). Biological functions unrelated to gene expression regulation were also proposed for lncRNAs. For example, the *NORAD* lncRNA was shown to assemble a topoisomerase complex critical for genome stability (Munschauer et al. 2018), while the X-linked *Firre* lncRNA is involved in chromatin super-loop formation on the inactive X chromosome (Hacisuleyman et al. 2014; Barutcu et al. 2018). Additional evidence that individual lncRNAs are undoubtedly biologically relevant comes from associations with human diseases, including cancer, as for the *SAMMSON* lncRNA (Vendramin et al. 2018).

Initial studies of lncRNA functionality generally asserted that biological functions are directly carried out by the transcribed RNA molecules. For some lncRNAs, this hypothesis was supported by thorough functional tests, including rescue experiments showing that phenotypic effects of lncRNA locus deletion can be reversed by expressing the lncRNAs in *trans* (Munschauer et al. 2018). Thus, for a subset of lncRNA loci, their biological function is undoubtedly achieved by the noncoding RNA molecule. However, in some cases, lncRNA function resides in the act of transcription at a given genomic location, rather than in the product of transcription (Latos et al. 2012). In other cases, biological functions are carried out by other elements embedded in the lncRNA genomic loci (Bassett et al. 2014). For example, transcription of *Linc-p21*, originally described as a *cis*-acting enhancer lncRNA, is not needed to regulate neighboring gene expression, which is instead controlled by multiple enhancer elements within the locus (Groff et al. 2016). Genetic engineering of multiple lncRNA loci in mouse likewise indicated that lncRNA transcripts are dispensable, and that gene expression regulation by lncRNA loci is instead achieved by the process of transcription and splicing, or by additional regulatory elements found in lncRNA promoters (Anderson et al. 2016; Engreitz et al. 2016). Furthermore, some attempts to look for lncRNA function through genetic engineering approaches showed that the tested lncRNA loci are altogether dispensable (Amândio et al. 2016; Zakany et al. 2017; Goudarzi et al. 2019). These recent observations signal a paradigm shift in lncRNA biology, as it is increasingly acknowledged that, even when phenotypic effects can be unambiguously mapped to lncRNA loci, they may not be driven by the lncRNA transcripts themselves.

Importantly, this new perspective on lncRNA biology had been predicted by evolutionary analyses, traditionally used to evaluate the functionality of diverse genomic elements (Haerty and Ponting 2014; Ulitsky 2016). Evolutionary studies of lncRNAs in vertebrates agree that selective constraint on lncRNA primary sequences is weak, though significantly above the genomic background (Ponjavic et al. 2007; Kutter et al. 2012; Necsulea et al. 2014; Washietl et al. 2014; Hezroni et al. 2015). These observations are compatible with the hypothesis that many of the lncRNAs detected with sensitive transcriptomics techniques may be nonfunctional noise (Ponjavic et al. 2007), or that their function is carried out by small conserved elements, such that the selective constraint signal on the entire lncRNA locus is overall weak (Ulitsky 2016). They also indicate that lncRNA functionality may not reside in the primary transcribed sequence. Indeed, mammalian lncRNA promoters show higher levels of sequence conservation, similar to protein-coding gene promoters (Necsulea et al. 2014), as expected if they carry out additional regulatory functions independently of the transcribed RNA molecule. Moreover, it was previously reported that, in multiexonic lncRNAs, splicing regulatory elements are more conserved than exonic sequences (Schüler et al. 2014; Haerty and Ponting 2015), in agreement with the recent finding that lncRNA splicing can contribute to neighboring gene regulation (Engreitz et al. 2016). Thus, detailed evolutionary analyses of lncRNA loci can bring insights into their functionality, and can help prioritize candidates for experimental validation.

Most comparative lncRNA studies were so far restricted to adult organ transcriptomes. These comparisons showed that lncRNAs are preferentially expressed in adult testes, during spermatogenesis (Soumillon et al. 2013). This process is characterized by a permissive chromatin environment, which can promote nonfunctional transcription (Soumillon et al. 2013). The resulting lncRNA data sets may thus be enriched in nonfunctional transcripts. Additional lines of evidence suggest that the search for functional lncRNAs should be extended beyond adult organ transcriptomes. For example, involvement in developmental phenotypes was proposed for many experimentally tested lncRNAs (Ulitsky et al. 2011; Grote et al. 2013; Sauvageau et al. 2013), and an enrichment for developmental transcription factor binding was reported for the promoters of conserved lncRNAs (Necsulea et al. 2014). These observations motivated us to add a temporal dimension to comparative lncRNA transcriptomics studies. Therefore, here, we characterize lncRNAs across species, organs, and developmental stages. We analyze the spatial and temporal expression patterns of protein-coding genes and lncRNAs, in conjunction with their evolutionary conservation. We find that, while lncRNAs are overall poorly conserved during evolution in terms of primary sequence or expression patterns, higher frequencies of constrained lncRNAs are observed in embryonic transcriptomes. For many of these loci, biological function may be RNA-independent, as the highest levels of sequence conservation are observed on promoter regions and on splice signals, rather than on lncRNA exonic sequence. Our results are thus compatible with unconventional, RNA-independent functions for evolutionarily conserved lncRNA loci, in particular, for those that are expressed during embryonic development.

## Results

### Comparative Transcriptomics across Species, Organs, and Developmental Stages

**Fig. 1.**
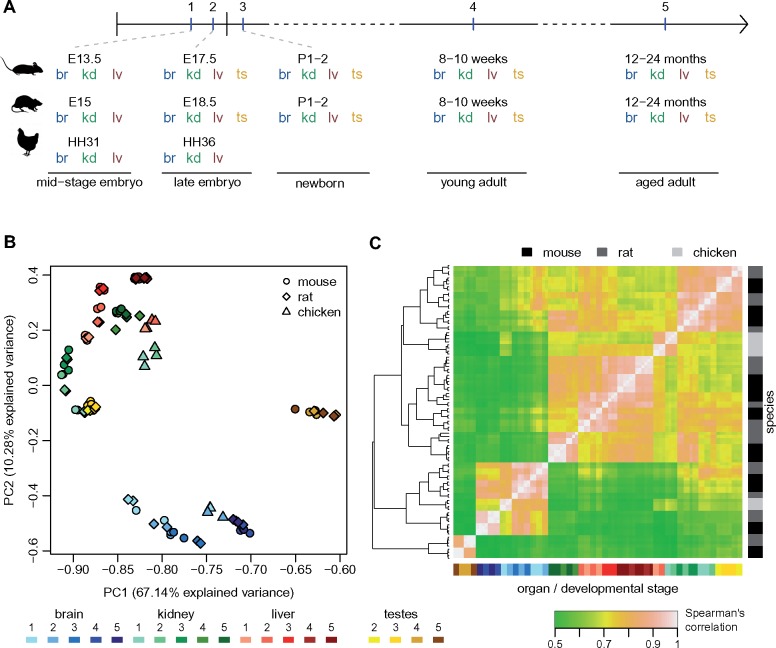
Comparative transcriptomics across species, organs, and developmental stages. (*A*) Experimental design. The developmental stages selected for mouse, rat, and chicken are marked on a horizontal axis. Organs sampled for each species and developmental stage are shown below. Abbreviations: br, brain; kd, kidney; lv, liver; ts, testes. (*B*) First factorial map of a principal component analysis, performed on log2-transformed TPM values, for 10,363 protein-coding genes with orthologs in mouse, rat, and chicken. Colors represent different organs and developmental stages, point shapes represent different species. (*C*) Hierarchical clustering, performed on a distance matrix derived from Spearman correlations between pairs of samples, for 10,363 protein-coding genes with orthologs in mouse, rat, and chicken. Organ and developmental stages are color-coded, shown below the heatmap. Species of origin is color-coded, shown on the right. Sample clustering is shown on the left.

To study protein-coding and lncRNA expression patterns across both developmental and evolutionary time, we generated strand-specific RNA-seq data for mouse and rat, for four major organs (brain, kidney, liver, and testes) and five developmental time points, including two embryonic stages, newborn, young, and aged adult individuals (fig. 1*A*;supplementary table 1, Supplementary Material online; and see Materials and Methods). The selected time points allow us to obtain a broad view of major organ ontogenesis and to capture drastic physiological changes during development (Theiler 1989). We chose to include in our study both young adult (8–10 weeks old) and aged adult individuals (12–24 months old), thus completing our overview of temporal patterns of gene expression variation. At the earliest embryonic stage (day 13.5 postconception for mouse, day 15 for rat), we dissected only the three somatic organs. Our experimental design for mouse and rat thus comprises 19 organ/developmental stage combinations. To obtain a broader evolutionary perspective, we generated comparable RNA-seq data for the chicken, for the two earliest developmental stages (fig. 1*A* andsupplementary table 1, Supplementary Material online). We generated between two and four biological replicates for each species/organ/developmental stage combination (supplementary table 1, Supplementary Material online). Additional RNA-seq samples from previous publications were included in the lncRNA annotation process, to increase detection sensitivity (supplementary table 2, Supplementary Material online).

The organs and developmental stages included in our study differ greatly in terms of their cellular composition diversity. To verify that our whole-organ RNA-seq data reflect cellular composition heterogeneity, we assessed the expression patterns of cell population markers derived from single-cell transcriptomics studies (Green et al. 2018; Tabula Muris Consortium 2018) in our samples (supplementary fig. 1, table 3, and methods, Supplementary Material online). This analysis confirms that our transcriptome collection reflects expected developmental patterns. For example, mature oligodendrocyte cell markers are systematically highly expressed in adult brain, while oligodendrocyte precursor markers are more highly expressed in the earliest developmental stages (supplementary fig. 1, Supplementary Material online). To further characterize our transcriptome collection, we sought to identify genes that could serve as markers for organ/developmental stage combinations. To do this, we selected genes that have narrow expression distributions, and for which maximum expression is observed in the same organ/developmental stage combination in mouse and rat (supplementary methods and table 4, Supplementary Material online). Gene ontology enrichment analyses for these lists of genes are coherent with the cellular composition and biological processes at work. Thus, genes involved in forebrain neuron differentiation are overrepresented in the midstage embryonic brain, while processes related to synaptic transmission are enriched among genes specifically expressed in adult brain (supplementary fig. 2, Supplementary Material online). In the kidney, the early developmental stages are enriched in genes involved in metanephric development (supplementary fig. 3, Supplementary Material online). The newborn liver stands out due to its strong enrichment in genes involved in immune response, while metabolic processes are overrepresented in the adult liver (supplementary fig. 4, Supplementary Material online). Embryonic testes samples express genes implicated in gamete generation and gene silencing by miRNAs, including the *Piwi-*like genes, while adult testes transcriptomes are dominated by genes involved in spermatogenesis (supplementary fig. 5, Supplementary Material online). These patterns confirm that our whole-organ transcriptome collection captures the cell composition changes and physiological transitions that occur during organ development.

### Developmental Expression Patterns Are Well Conserved among Species for Protein-Coding Genes

To gain a first glimpse into the evolution of developmental gene expression patterns, we performed a principal component analysis (PCA) for 10,363 protein-coding genes shared among mouse, rat, and chicken (fig. 1*B* andsupplementary methods, Supplementary Material online). This analysis revealed that the main source of gene expression variability among species, organs, and developmental stages is the distinction between adult and aged testes and the other samples, which are separated on the first PCA axis (fig. 1*B*). In contrast, embryonic and newborn testes are grouped with kidney samples from similar developmental stages, in agreement with the common developmental origin of the kidney and the gonads (Nel-Themaat et al. 2010). The first axis of the PCA, which explains 67% of the total expression variance, also correlates with the developmental stage for the brain: samples derived from adult and aged individuals have higher coordinates on this axis than embryonic and newborn samples (fig. 1*B* andsupplementary fig. 6, Supplementary Material online, Kruskal–Wallis test *P* value 0.003). The second PCA axis (10% explained variability) mainly reflects the difference between brain and the other organs, but is also associated with the developmental stage for kidney and liver (fig. 1*B* andsupplementary fig. 6, Supplementary Material online, Kruskal–Wallis test *P* value 4e^−4^ for kidney, 4e^−5^ for liver). However, we note that the association between PC2 and developmental stages for kidney and liver may be confounded by differences in RNA degradation among developmental stages for these organs (supplementary methods and fig. 6, Supplementary Material online).

Although mouse and rat samples are almost undistinguishable on the PCA factorial map, there is considerably higher expression divergence between chicken and the two rodent species (fig. 1*B*). However, differences among major organs are stronger than differences among species, even at these broad evolutionary distances: brain samples all cluster together, irrespective of the species of origin, and are clearly separated from kidney and liver samples on the second PCA axis (fig. 1*B*). These patterns of gene expression variations are confirmed by a hierarchical clustering analysis based on Spearman’s correlation coefficients between pairs of samples (fig. 1*C*). The strongest clustering is observed for adult and aged testes samples, followed by brain samples (fig. 1*C*).

The grouping among samples derived from similar organs and developmental stages, irrespective of the species of origin, is stronger for genes that are associated with embryonic development and with gene expression regulation (supplementary methods and fig. 6*C* and *D*, Supplementary Material online). For this set of genes, both the PCA and the hierarchical clustering analysis show a near-perfect separation of organs and developmental stages, for all three species (supplementary fig. 6*C* and *D*, Supplementary Material online). Chicken samples, which cluster apart from rodent samples in whole transcriptome analyses, are now grouped with the corresponding organs and developmental stages from mouse and rat. Our transcriptome collection can thus reveal highly conserved expression patterns for regulators of embryonic development, across amniotes.

### Variations in Transcriptome Complexity among Organs and Developmental Stages

We next sought to assess the transcriptome complexity in different organs across developmental stages. To predict lncRNAs, we used the RNA-seq data to reconstruct gene models with StringTie (Pertea et al. 2015), building on existing genomic annotations (Cunningham et al. 2019). We verified the protein-coding potential of newly annotated transcripts, based on the codon substitution frequency score (Lin et al. 2007, 2011) and on sequence similarity with known proteins, and we applied a stringent series of filters to reduce contaminations from unannotated protein-coding UTRs and other artifacts (see Materials and Methods). We thus obtain a total of 18,858 candidate lncRNAs in the mouse, 20,159 in the rat and 5,496 in the chicken, including both newly annotated and previously known lncRNAs transcribed in our samples (supplementary data set 1, Supplementary Material online). The relative sizes of each species’ lncRNA repertoires are consistent with previous studies (Necsulea et al. 2014; Sarropoulos et al. 2019). We note however that our power to detect lncRNAs in chicken is limited, due to the narrower organ and developmental stage sampling in this species (supplementary tables 1 and 2, Supplementary Material online). Most candidate lncRNAs are expressed at very low levels. When imposing a minimum normalized expression level (transcript per million or TPM) of 1, in at least one sample, the numbers of candidate lncRNAs falls to 12,199, 15,319, and 2,892 in the mouse, rat, and chicken, respectively (supplementary data sets 2 and 3, Supplementary Material online).

**Fig. 2.**
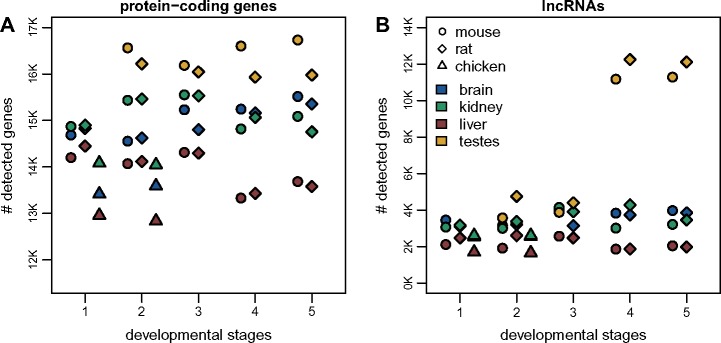
Transcriptome complexity in different species, organs, and developmental stages. (*A*) Number of protein-coding genes supported by at least ten uniquely mapped reads in each sample, after read resampling to homogenize coverage (supplementary methods, Supplementary Material online). Colors represent different organs, point shapes represent different species. Developmental stages are indicated by numeric labels, 1–5, on the *x* axis. We analyzed a total of 19,356 protein-coding genes in the mouse, 19,274 in the rat, and 15,509 in the chicken. (*B*) Number of lncRNAs supported by at least ten uniquely mapped reads in each sample, after read resampling to homogenize coverage. We analyzed a total of 18,858 candidate lncRNAs in the mouse, 20,159 in the rat, and 5,496 in the chicken.

The differences in lncRNA content among species may be affected by RNA-seq read coverage and sample distribution, as well as genome sequence and annotation quality. To correct for the effect of RNA-seq read coverage, we down-sampled the RNA-seq data to obtain the same number of uniquely mapped reads for each organ/developmental stage combination within each species (supplementary methods, Supplementary Material online). After this procedure, the number of detectable protein-coding genes (supported by at least ten uniquely mapped reads) still shows broad variations among organs and developmental stages, with the highest numbers of genes detected in the testes, for all time points (fig. 2*A*). Large numbers of protein-coding genes (between ∼12,800 and 16,700) are detected in all samples. In contrast, for lncRNAs, the pattern is much more striking: the young and aged adult testes express between 11,000 and 12,000 lncRNAs, in both mouse and rat, while in somatic organs and earlier developmental stages, we can detect only between 1,800 and 4,800 lncRNAs (fig. 2*B*). This observation is in agreement with previous findings indicating that during spermatogenesis the chromatin environment is highly permissive to transcription (Soumillon et al. 2013).

### Spatial and Temporal Expression Patterns for Protein-Coding Genes and lncRNAs

**Fig. 3.**
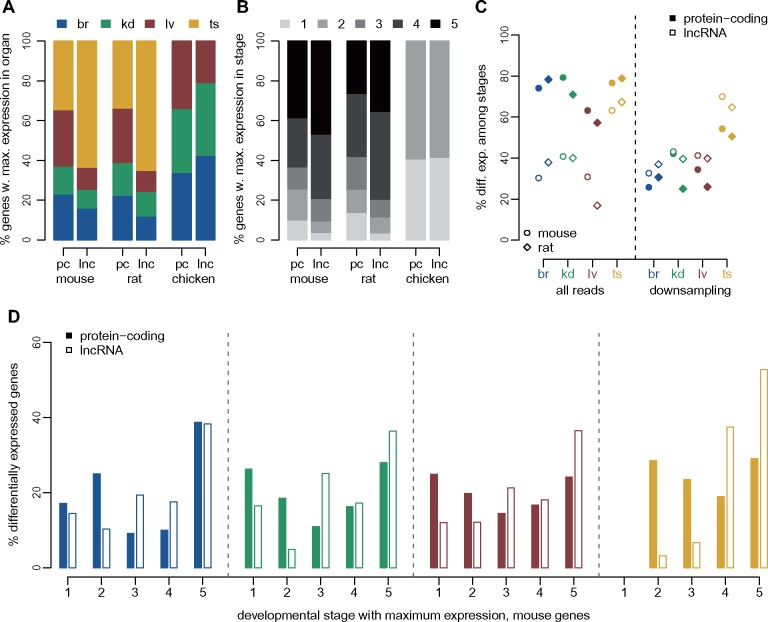
Different expression patterns for protein-coding genes and lncRNAs. (*A*) Distribution of the organ in which maximum expression is observed, for protein-coding genes (pc) and lncRNAs (lnc), for mouse, rat, and chicken. Organs are color-coded, shown above the plot. We defined the sample in which maximum expression is observed based on an average expression values across replicates, for each organ/developmental stage combination (supplementary methods, Supplementary Material online). (*B*) Distribution of the developmental stage in which maximum expression is observed, for protein-coding genes and lncRNAs, for mouse, rat, and chicken. Developmental stages are color-coded, shown above the plot. (*C*) Percentage of protein-coding and lncRNA genes that are significantly (FDR<0.01) differentially expressed (DE) among developmental stages, with respect to the total number of genes tested for each organ. Left panel: differential expression analysis performed with all RNA-seq reads. Right panel: differential expression analysis performed after down-sampling read counts for protein-coding genes, to match those of lncRNAs (see Materials and Methods). (*D*) Distribution of the developmental stage in which maximum expression is observed, for protein-coding genes and lncRNAs that are significantly DE (FDR<0.01) in each organ, for the mouse. Percentages are computed with respect to the total number of DE genes in each organ and gene class.

We next compared spatial and temporal expression patterns between protein-coding genes and lncRNAs. In agreement with previous findings (Soumillon et al. 2013), we show that lncRNAs are overwhelmingly preferentially expressed in the testes (fig. 3*A*). Indeed, more than 60% of lncRNAs reach their maximum expression level in this organ, compared with less than 35% of protein-coding genes, for both mouse and rat (fig. 3*A*; χ^2^ test *P* value <1e^−10^). Almost 80% of lncRNAs are preferentially expressed in young or aged adult samples, which is significantly higher than the fraction observed for protein-coding genes (<65%, χ^2^ test *P* value <1e^−10^, fig. 3*B*).

We found that between 57% and 80% of protein-coding genes are significantly differentially expressed (DE) among developmental stages, at a false discovery rate (FDR) <1%, in each organ and species (fig. 3*C* and supplementary data set 4, Supplementary Material online). The proportions of DE lncRNAs are significantly lower than the proportions of DE protein-coding genes in somatic organs, between 17% and 41% (χ^2^ test, *P* value <1e^−10^). In the testes, we observed higher proportions of DE lncRNAs (63% in mouse and 67% in rat), but these values were still significantly lower than those observed for protein-coding genes (77% in mouse and 79% in rat; χ^2^ test, *P* value <1e^−10^; fig. 3*C*). We suspected that the lower proportion of DE lncRNAs could be due to their low expression levels, as total read counts affect the sensitivity of DE tests (Anders and Huber 2010). Indeed, lncRNAs are expressed at much lower levels than protein-coding genes (supplementary fig.7, Supplementary Material online). To control for this, we down-sampled the read counts observed for protein-coding genes, bringing them to the same average counts as lncRNAs but preserving relative gene abundance (see Materials and Methods). Strikingly, after down-sampling, we observe higher proportions of DE loci for lncRNAs compared with protein-coding genes (fig. 3*C*). The differences are statistically significant (χ^2^ test, *P* value <1e^−10^) in all but one species/organ combination (mouse kidney, χ^2^ test, *P* value 0.15). We also observed that the expression amplitude among developmental stages are more important for lncRNAs than for protein-coding genes (Wilcoxon test, *P* value <1e^−10^, supplementary fig. 8*A*, Supplementary Material online), as expected given the lower lncRNA expression levels, which preclude detecting subtle expression shifts among time points. Finally, we observe that the developmental stage with maximum expression is generally different between protein-coding genes and lncRNAs, even when considering genes that are significantly DE among stages. For all organs, DE lncRNAs tend to show highest expression levels in the young and aged adults, while DE protein-coding genes are more homogeneously distributed among developmental stages (χ^2^ test, *P* value <1e^−10^, fig. 3*D* and supplementary fig. 8*B*, Supplementary Material online).

Similar conclusions are reached when performing DE analyses between consecutive time points (supplementary fig. 9 and data set 4, Supplementary Material online). For both protein-coding genes and lncRNAs, the strongest expression changes are observed between newborn and young adult individuals. Almost 10,000 lncRNAs are significantly upregulated between newborn and young adult testes, confirming the strong enrichment for lncRNAs during spermatogenesis (supplementary fig. 9, Supplementary Material online). As expected, the lowest numbers of DE genes are observed at the transition between young and aged adult organs. At this time point, we observe more changes for the rat than for the mouse, potentially due to a higher proportion of immune cell infiltrates in the rat aged organ samples. Genes associated with antigen processing and presentation tend to be expressed at higher levels in aged adults than in young adults, for mouse kidney, rat brain, and liver (supplementary data set 4, Supplementary Material online).

### Stronger Selective Constraint on lncRNAs Expressed Earlier in Development

We next analyzed the long-term evolutionary sequence conservation for lncRNAs, in conjunction with their spatio-temporal expression patterns (supplementary table 5, Supplementary Material online). We used the PhastCons score (Siepel et al. 2005) for placental mammals (Casper et al. 2018), to assess sequence conservation for various aspects of mouse lncRNAs: exons, promoters (defined as 400 bp regions upstream of the transcription start site), splice sites (first and last two bases of the introns). As approximately 20% of lncRNAs overlap with exonic regions on the opposite strand (supplementary data set 1, Supplementary Material online), we masked exonic regions from other genes before evaluating sequence conservation.

As previously observed (Ponjavic et al. 2007; Haerty and Ponting 2013), exonic and splice site sequence conservation is much lower for lncRNAs (median exonic score 0.094, median splice site score 0.075) than for protein-coding genes (median exonic score 0.42, median splice site score 0.85, Wilcoxon test *P* value <1e^−10^, supplementary fig. 10, Supplementary Material online). Exonic lncRNA conservation scores are significantly above the conservation observed for intergenic regions genome-wide (median score 0.076, Wilcoxon test, *P* value <1e^−10^, supplementary fig. 10, Supplementary Material online). Interestingly, intergenic regions found in the vicinity of lncRNA loci (supplementary methods, Supplementary Material online) had slightly lower conservation scores than all intergenic regions, on an average (median 0.072, Wilcoxon test, *P* value <1e^−6^, supplementary fig. 10, Supplementary Material online). Promoter conservation levels are more comparable between protein-coding genes (median score 0.17) and lncRNAs (median score 0.08), though still significantly higher for the former (Wilcoxon test, *P* value <1e^−10^, supplementary fig. 10, Supplementary Material online). Among lncRNA classes, the highest levels of promoter sequence conservation (median 0.14) are observed for bidirectional promoters shared with protein-coding genes (supplementary fig. 10, Supplementary Material online).

**Fig. 4.**
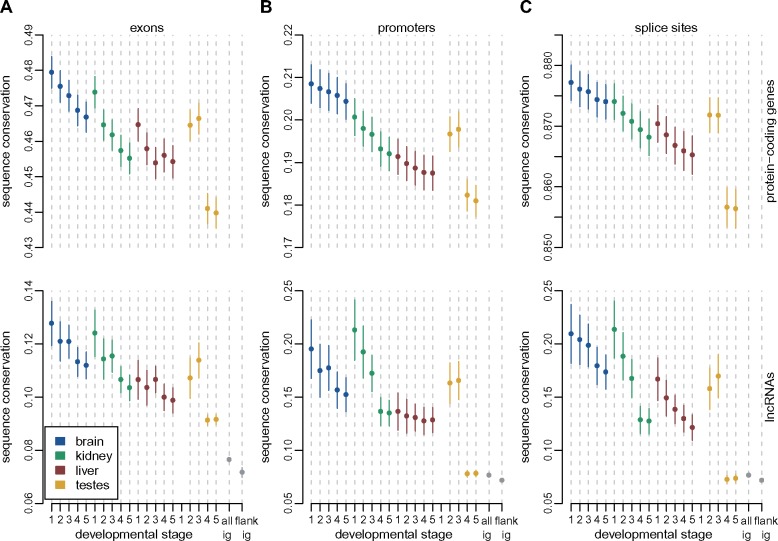
Increased levels of long-term sequence conservation for lncRNAs expressed early in development. (*A*) Sequence conservation scores (PhastCons scores, placental mammals) for protein-coding and lncRNAs exonic regions, for subsets of genes expressed above noise levels (TPM≥1) in each organ and developmental stage. Dots represent medians, vertical bars represent 95% confidence intervals. Numbers of analyzed genes are provided in supplementary table 5, Supplementary Material online. Organs are color-coded; developmental stages are indicated (numbers 1–5) on the *x* axis. The gray dots and vertical bars represent the median value and 95% confidence interval for all intergenic regions, genome-wide, or for intergenic regions flanking lncRNA loci (supplementary methods, Supplementary Material online). (*B*) Same as (*A*), for promoter regions (400 bp upstream of transcription start sites). Exonic sequences were masked before assessing conservation. (*C*) Same as (*B*), for splice sites (first and last two bases of each intron).

We next analyzed sets of protein-coding genes and lncRNAs that are expressed above noise levels (TPM≥1, averaged across all biological replicates) in each organ/developmental stage combination (supplementary table 6, Supplementary Material online). For all examined regions and for both categories of genes, the spatio-temporal expression pattern is associated with the level of sequence conservation. Globally, sequence conservation is higher for genes that are expressed earlier in development than for genes expressed later in development, and reaches its lowest values for genes expressed in adult and aged testes (fig. 4). For exonic sequences and splice sites, the amount of sequence conservation is significantly lower for lncRNAs than for protein-coding genes, irrespective of the organ and developmental stage in which they are expressed (Wilcoxon test, *P* value <1e^−10^, fig. 4*A* and *C*). However, for promoter regions, the differences between the two gene categories are weaker, and are not statistically significant for the midstage embryonic brain (median 0.21 for protein-coding genes, 0.20 for lncRNAs, Wilcoxon test, *P* value 0.08) and kidney (median 0.20 for protein-coding genes, 0.21 for lncRNAs, Wilcoxon test, *P* value 0.76), and for the late embryonic kidney (median 0.20 for protein-coding genes, 0.19 for lncRNAs, Wilcoxon test, *P* value 0.15). As noted before, the highest levels of lncRNA promoter conservation are observed for lncRNAs that have bidirectional promoters shared with protein-coding genes or other noncoding loci (supplementary fig. 11*A*–*C*, Supplementary Material online).

Finally, we asked whether the highest level of evolutionary sequence conservation is seen at exons, promoter, or splice site regions, for lncRNA loci taken individually. Here again, the answer depends on the expression pattern: for lncRNAs detected in somatic organs and in the developing testes, there is significantly higher conservation for promoters than for exons (Wilcoxon test, *P* value <1e^−3^ for all organ/developmental stage combinations, supplementary fig. 11*D* and *E*, Supplementary Material online). We also observe significantly higher conservation for splice sites than for exons (Wilcoxon test, *P* value <0.005), in all samples except aged liver (Wilcoxon test, *P* value 0.052). However, when we consider lncRNAs that are expressed above noise levels in the young and aged adult testes (which constitute the great majority of loci), the conservation scores are slightly but significantly higher for exons than for promoters or splice sites (Wilcoxon test, *P* value <1e^−9^, supplementary fig. 11*D* and *E*, Supplementary Material online).

### Detection of Homologous lncRNAs across Species

**Fig. 5.**
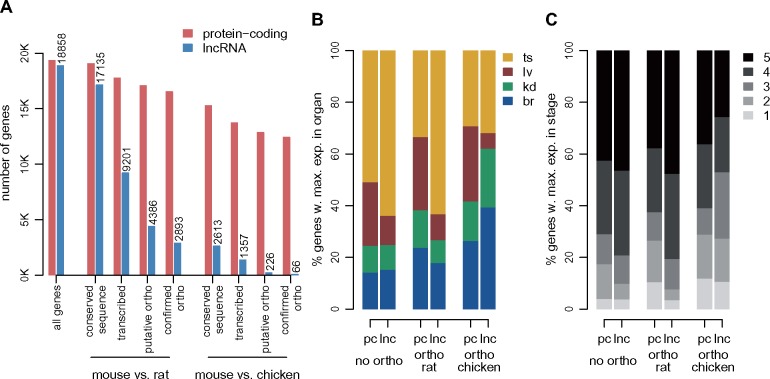
Orthologous lncRNA families for mouse, rat, and chicken. (*A*) Number of mouse protein-coding genes and lncRNAs in different classes of evolutionary conservation. From left to right: all loci, loci with conserved sequence in the rat, loci for which transcription could be detected (at least ten unique reads) in predicted orthologous locus in the rat, loci with predicted 1-to-1 orthologs, loci for which the predicted ortholog belonged to the same class (protein-coding or lncRNA) in the rat, loci with conserved sequence in the chicken, loci for which transcription could be detected (at least ten unique reads) in predicted orthologous locus in the chicken, loci with predicted 1-to-1 orthologs, loci for which the predicted ortholog belonged to the same class (protein-coding or lncRNA) in the chicken. We analyze 19,356 protein-coding genes and 18,858 candidate lncRNAs in the mouse. (*B*) Distribution of the organ in which maximum expression is observed, for mouse protein-coding and lncRNA genes that have no orthologs in the rat or chicken, for genes with orthologs in the rat and for genes with orthologs in chicken. The sample in which maximum expression is observed is computed based on an average expression values across biological replicates, for each organ/developmental stage combination (supplementary methods, Supplementary Material online). (*C*) Same as (*B*), for the distribution of the developmental stage in which maximum expression is observed.

We next sought to assess the conservation of lncRNA repertoires in mouse, rat, and chicken. We detected lncRNA separately in each species, using only RNA-seq data and existing genome annotations, as previously suggested (Hezroni et al. 2015). We then searched for putative 1-to-1 orthologous lncRNAs between species using precomputed whole-genome alignments as a guide (see Materials and Methods), to increase the sensitivity of orthologous gene detection in the presence of rapid sequence evolution (Washietl et al. 2014). The orthologous lncRNA detection procedure involves several steps, including the identification of putative homologous (projected) loci across species, filtering to remove large-scale structural changes in the loci, and intersection with predicted loci in the target species (see Materials and Methods). As illustrated in figure 5, for comparisons between rodents the extent of sequence divergence is low enough that >90% of 18,858 lncRNA loci are successfully projected from mouse to rat (fig. 5*A*, supplementary data set 5, Supplementary Material online). However, only 54% of projected loci have detectable transcription in the target species (at least ten uniquely mapped reads). Only 23% of mouse lncRNA loci have predicted 1-to-1 orthologs in the rat, and only 15% are orthologous to confirmed lncRNA loci in the rat (fig. 5*A*, supplementary data set 5, Supplementary Material online). The 1,493 mouse lncRNAs that have non-lncRNAs orthologs in the rat are generally matched with loci discarded because of low read coverage, minimum exonic length, or distance to protein-coding genes (supplementary data set 5, Supplementary Material online). Cases of lncRNA-protein-coding orthologs are rare at this evolutionary distance (supplementary data set 5, Supplementary Material online), and they may stem from gene classification errors. We note that orthologous lncRNA gene structures are highly divergent across species, in terms of exonic length or number of exons (supplementary fig. 12, Supplementary Material online). At larger evolutionary distances, the rate of sequence evolution is the main factor hampering detection of orthologous lncRNAs. Only 2,613 (14%) of mouse lncRNAs could be projected on the chicken genome, and after subsequent filtering, we detect only 66 mouse–chicken lncRNA orthologs, and 30 lncRNAs with orthologs in all three species (supplementary data set 5 and table 7, Supplementary Material online).

Conserved lncRNAs differ from species-specific lncRNAs in terms of expression patterns. Although only subtle differences can be observed when comparing mouse–rat orthologous lncRNAs to the mouse-specific lncRNA set, lncRNAs that are conserved between mouse and chicken are enriched in somatic organs and early developmental stages (fig. 5*B* and *C*). For example, only 15% of mouse-specific lncRNAs reach their maximum expression in the brain, which is significantly lower than the observed proportion for mouse lncRNAs with orthologs in rat (18%, χ^2^ test, *P* value 3e^−4^) and for mouse lncRNAs with orthologs in the chicken (39%, χ^2^ test, *P* value 1.5e^−7^). Likewise, while only 9.9% of mouse-specific lncRNAs have their highest level of expression in one of the two embryonic stages, this proportion is significantly higher for lncRNAs with orthologs in the chicken (27%, χ^2^ test, *P* value 0.002). We note however that these results may be affected by our narrower sampling for the chicken, which is biased toward embryonic organs, although we did include data from adult organs for this species (supplementary table 2, Supplementary Material online).

### Patterns of lncRNA Expression Variation across Species, Organs, and Developmental Stages

**Fig. 6.**
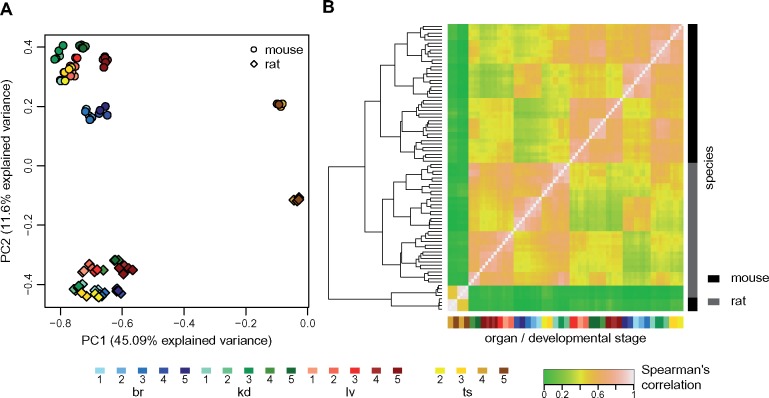
Global comparison of lncRNA expression patterns across species. (*A*) First factorial map of a principal component analysis, performed on log2-transformed TPM values, for 2,893 orthologous lncRNAs between mouse and rat. Colors represent different organs and developmental stages, point types represent species. (*B*) Hierarchical clustering, performed on a distance matrix derived from Spearman correlations between pairs of samples, for 2,893 orthologous lncRNAs between mouse and rat. Organ and developmental stages are shown below the heatmap. Species of origin is shown on the right. Sample clustering is shown on the left.

We next assessed the global patterns of expression variation across species, organs, and developmental stages, for predicted mouse–rat lncRNA orthologs (supplementary data set 6, Supplementary Material online). As for protein-coding genes, the main source of variability in a PCA performed on lncRNA expression levels is the difference between adult and aged testes and the other samples (fig. 6*A*). However, for lncRNAs, samples cluster according to the species of origin already on the second factorial axis (11.6% explained variance), confirming that lncRNA expression patterns evolve rapidly. Overall, differences between organs and developmental stages are less striking for lncRNAs, compared with differences between species (fig. 6*A*). This pattern is also visible on a hierarchical clustering analysis (performed on distances derived from Spearman’s correlation coefficient): in contrast with what is observed for protein-coding genes, for lncRNAs samples generally cluster by species, with the exception of young and aged adult testes, which are robustly grouped (fig. 6*B*).

**Fig. 7.**
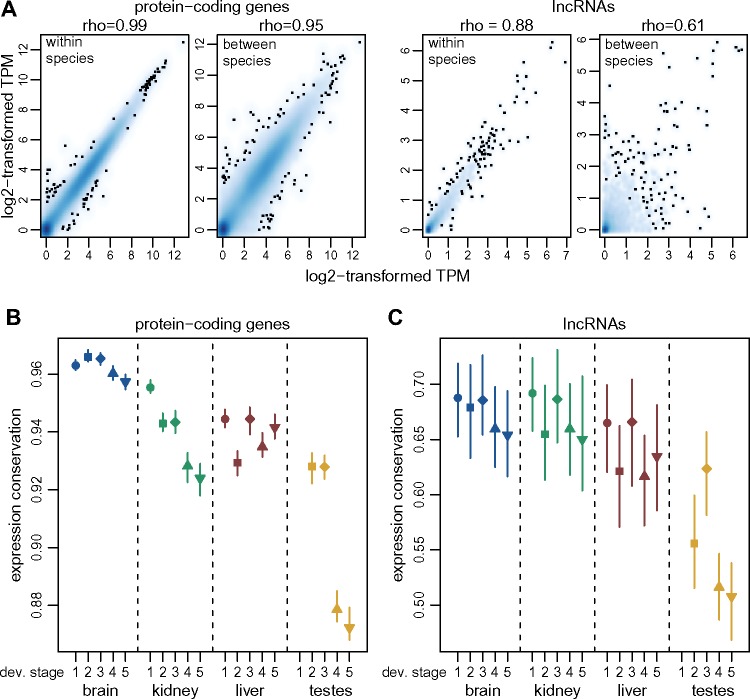
Global estimates of expression conservation across organs and developmental stages. (*A*) Example of between-species and within-species variation of expression levels, for protein-coding genes (left) and lncRNAs (right), for orthologous genes between mouse and rat, for the midstage embryonic brain. Spearman’s correlation coefficients (rho) are shown above each plot. We show a smoothed color density representation of the scatterplots, obtained through a 2D kernel density estimate (smoothScatter function in R). (*B*) Expression conservation index, defined as the ratio of the between-species and the within-species expression level correlation coefficients, for protein-coding genes, for each organ and developmental stage. The vertical segments represent minimum and maximum values obtained from 100 bootstrap replicates. We analyzed 15,931 pairs of orthologous protein-coding genes. (*C*) Same as (*B*), for lncRNAs. We analyzed 2,893 orthologous mouse and rat lncRNAs.

The higher rates of lncRNA expression evolution are also visible when analyzing within-species variations, through comparisons across biological replicates (fig. 7*A*). We sought to measure the selective pressures acting on expression patterns by contrasting between-species and within-species variations, in the spirit of a classical approach for coding sequences (McDonald and Kreitman 1991). We constructed an expression conservation index by dividing the between-species and the within-species Spearman’s correlation coefficient, computed on all genes from a category, for a given organ/developmental stage combination (supplementary methods, Supplementary Material online). The resulting values are very high for protein-coding genes, in particular for the brain and the midstage embryonic kidney, where the expression conservation scores are >0.95. However, there is significant less conservation between species for the adult and aged testes (expression conservation score ∼0.88, bootstrap *P* value <0.01, fig. 7*B*). For lncRNAs, expression conservation values vary between 0.5 and 0.7, significantly lower than for protein-coding genes (bootstrap *P* value <0.01). The lowest conservation scores are observed for young and aged adult testes (fig. 7*C*).

### Parallel Patterns of Temporal Expression Variation for Mouse and Rat lncRNAs

**Fig. 8.**
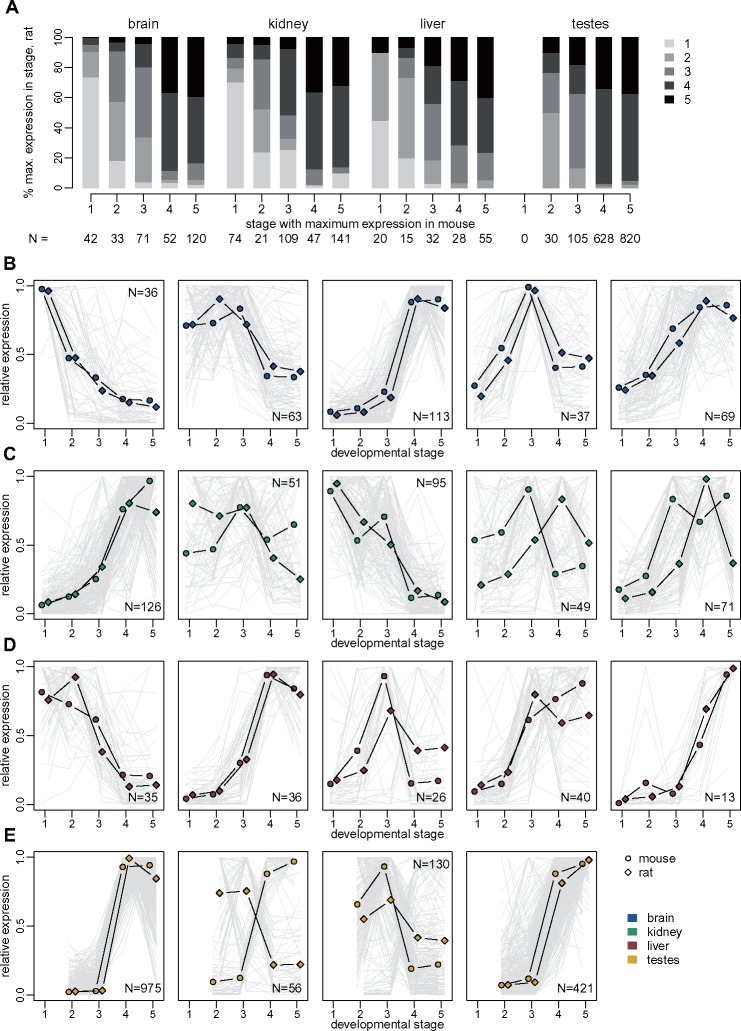
Conservation of developmental expression patterns of differentially expressed lncRNAs. (*A*) Comparison of the developmental stage in which maximum expression is observed, for orthologous lncRNAs that are significantly differentially expressed (FDR<0.01) among developmental stages, for both mouse and rat. The sample in which maximum expression is observed is computed based on an average expression values across biological replicates, for each organ/developmental stage combination (supplementary methods, Supplementary Material online). Genes are divided into classes based on the developmental stage where maximum expression is observed in mouse organs (*x* axis). The *y* axis represents the percentage of orthologous genes that reach maximum expression in each developmental stage, in the rat. Numbers of analyzed genes are shown below the plot. (*B*) Expression profiles of orthologous lncRNAs that are significantly differentially expressed (FDR<0.01) among developmental stages, for both mouse and rat, in the brain. TPM values were averaged across replicates and normalized by dividing by the maximum, for each species. The resulting relative expression profiles were combined across species and clustered with the K-means algorithm. Dots represent the average profiles of the genes belonging to each cluster. Gray lines represent profiles of individual genes from a cluster. Numbers of genes in each cluster are shown in the plot. (*C*) Same as (*B*), for the kidney. (*D*) Same as (*B*), for the liver. (*E*) Same as (*B*), for the testes. For this organ, we searched for only four clusters with the K-means algorithm.

We delved deeper into the evolutionary comparison of protein-coding genes and lncRNA expression patterns, by asking whether temporal expression variations are shared between species. Several hundred orthologous lncRNAs are DE (FDR < 0.01) in both mouse and rat, in each organ (minimum 150 in liver, maximum 1,583 in testes, fig. 8*A*). Likewise, between 6,775 (in liver) and 10,608 (in testes) protein-coding genes are DE in both species (supplementary fig. 13, Supplementary Material online). Overall, shared DE lncRNAs show similar patterns of variation among developmental stages in mouse and rat, reaching their maximum expression in the same (or close) developmental stages (fig. 8*A*). For example, out of 42 lncRNAs that are DE in mouse brain and reach their maximum expression in the midstage embryo, 31 (74%) reach their maximum expression in the corresponding stage in the rat (fig. 8*A*). We clustered the relative expression profiles using the K-means algorithm (supplementary methods, Supplementary Material online). Although individual gene trajectories show variations between species, the average expression profiles within each K-means cluster are generally similar between mouse and rat (fig. 8*B*–*E* and supplementary fig. 13, Supplementary Material online). This is particularly striking for the brain, where all five lncRNA clusters show similar average expression profiles for the two species (fig. 8*B*). Greater differences between species are observed in other organs, such as the kidney, where two out of five clusters (120 genes in total, that is 31% of shared DE lncRNAs in kidney) have average expression profiles that reach their maximum in different stages in mouse and rat (fig. 8*C*). The promoters of shared DE lncRNAs in each cluster contain transcription factor binding sites that are coherent with the expression profile of the cluster, such as brain homeobox POU3F2/BRN2 binding sites for the first K-means cluster in the brain, which has maximum expression in the midstage embryo (supplementary table 8, Supplementary Material online). We note that transcription factor binding site enrichments are generally not statistically significant for lncRNAs, partly due to the low gene counts in each cluster.

The testis is the only organ where opposite K-mean cluster expression profiles are observed in the two species (increasing with time in mouse and decreasing in rat, or vice versa). For lncRNAs, this occurs for one of the four detected clusters, containing 56 lncRNAs (3.5% of all shared DE lncRNAs in this organ, fig. 8*E*). For protein-coding genes, opposite average profiles are observed for two out of four clusters, comprising 1,182 and 1,509 genes, that is, 25% of all shared testes-DE protein-coding genes (supplementary fig. 13, Supplementary Material online). These clusters do not stand out in terms of transcription factor binding site (supplementary table 8, Supplementary Material online) or gene ontology enrichment (supplementary data set 4, Supplementary Material online). This pattern confirms previous reports of rapid expression evolution in the adult testes (Brawand et al. 2011), and extends them by showing that patterns of variations among developmental stages are often species-specific in the testes, for protein-coding genes.

### Evolutionary Divergence of Individual lncRNA Expression Profiles

**Fig. 9.**
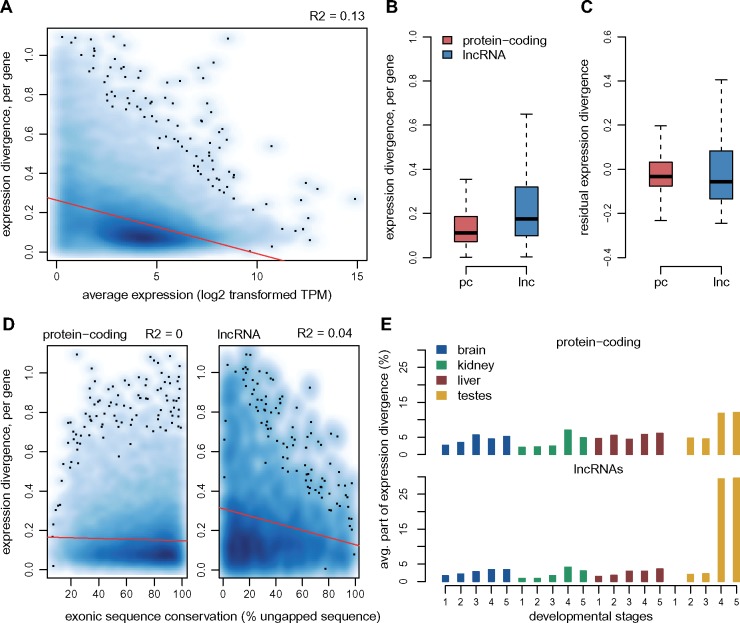
*Per*-gene estimates of expression pattern divergence between species. (*A*) Relationship between the per-gene expression divergence measure (Euclidean distance of relative expression profiles among organs/stages, between mouse and rat), and the average expression values (log2-transformed TPM) across all mouse and rat samples. We show a smoothed color density representation of the scatterplots, obtained through a 2D kernel density estimate (smoothScatter function in R). Red line, linear regression. (*B*) Distribution of the expression divergence value for all protein-coding and lncRNA genes with predicted 1-to-1 orthologs in mouse and rat. (*C*) Distribution of the residual expression divergence values, after regressing the average expression level, for protein-coding genes and lncRNAs. (*D*) Relationship between expression divergence and exonic sequence conservation (% exonic sequence aligned without gaps between mouse and rat), for protein-coding genes and lncRNAs. (*E*) Average contribution of each organ/developmental stage combination to expression divergence, for protein-coding genes and lncRNAs.

To further quantify lncRNA expression differences between species, we measured the Euclidean distance between relative expression profiles (average TPM values across biological replicates, normalized by dividing by the sum of all values for a gene, for each species), for mouse and rat orthologs (supplementary methods, data set 7, and table 9, Supplementary Material online). The resulting expression divergence values correlate negatively with the average expression level (*R*^2^ 0.13, *t*-test *P* value <1e^−10^, fig. 9*A*), as expected given that abundance estimation is less reliable for weakly expressed genes. Although the raw expression divergence values are significantly higher for lncRNAs (median 0.18) than for protein-coding genes (median 0.11, Wilcoxon test *P* value <1e^−10^, fig. 9*B*), this is largely due to the low lncRNA expression levels. Indeed, the effect disappears when analyzing the residual expression divergence after regressing the expression level (median value −0.03 for protein-coding genes, −0.06 for lncRNAs, Wilcoxon test <1e^−10^, fig. 9*C*). These patterns remain true when analyzing separately protein-coding and lncRNAs with different types of promoters, bidirectional, or unidirectional (supplementary fig. 14*A*, Supplementary Material online). For lncRNAs, we also observe a weak negative correlation between expression divergence and the extent of gene structure conservation (R^2^ 0.04, *t*-test *P* value <1e^−10^, fig. 9*D*). We measured the relative contribution of each organ/developmental stage to the expression divergence estimate (fig. 9*E*). For both protein-coding genes and lncRNAs, by far the highest contributors are the young adult and aged testes samples, which are responsible for almost 30% of the lncRNA expression divergence (fig. 9*E*). This is visible in the expression patterns of the two protein-coding and lncRNA genes with the highest residual expression divergence: the lncRNA expression divergence is mostly due to changes in adult testes, while more complex expression pattern changes seem to have occurred for the protein-coding genes (supplementary fig. 14, Supplementary Material online). The most divergent protein-coding genes are enriched in functions related to immunity (supplementary data set 7, Supplementary Material online), suggesting that differences in immune cell infiltrates among species could be responsible for these extreme cases of expression pattern divergence.

### Candidate Species-Specific lncRNAs

We next investigated the most extreme cases of expression divergence: situations where expression can be robustly detected in one species, but not in the other one, despite almost perfect sequence alignment (supplementary methods, Supplementary Material online). We selected lncRNA loci that were supported by at least 100 uniquely mapped reads in one species, with no reads detected in the predicted homologous region in the other species. With this convention, we obtain 1,041 candidate mouse-specific and 1,646 candidate rat-specific loci (supplementary data set 8, Supplementary Material online). These lists include striking examples, such as the region downstream of the *Fzd4* protein-coding gene, which contains a mouse-specific and a rat-specific lncRNA candidate, each perfectly aligned in the other species (supplementary fig. 15, Supplementary Material online). Candidate species-specific lncRNAs are more frequently associated with predicted enhancers than orthologous lncRNAs (52% vs. 33%, χ^2^ test, *P* value <1e^−10^), are less often spliced (56% vs. 61%, χ^2^ test *P* value 1.6e^−3^) and associated with bidirectional promoters (24% vs. 61%, χ^2^ test, *P* value <1e^−10^, supplementary fig. 16, Supplementary Material online). Moreover, we could confirm that their presence is associated with increased expression divergence in the neighboring genes. To test this, we selected species-specific and orthologous lncRNAs that are transcribed from bidirectional promoters shared with protein-coding genes, and evaluated the expression divergence of their protein-coding neighbors (supplementary fig. 16*D* and *E*, Supplementary Material online). Though the difference is subtle, genes that are close to species-specific lncRNAs have significantly higher expression divergence than the ones that have conserved lncRNA neighbors, even after correcting for expression levels (Wilcoxon test, *P* value <1e^−3^). It thus seems that expression changes that led to the species-specific lncRNA transcription extend beyond the lncRNA locus and affect neighboring genes, as previously proposed (Kutter et al. 2012).

## Discussion

### Comparative Transcriptomics across Species, Organs, and Developmental Stages

More than a decade after the publication of the first genome-wide lncRNA data sets (Guttman et al. 2009; Khalil et al. 2009), the debate regarding their functionality is still not settled. Evolutionary approaches provide important tools to assess biological functionality (Haerty and Ponting 2014), and they have been already successfully applied to lncRNAs. However, most large-scale comparative transcriptomics studies available so far (Kutter et al. 2012; Necsulea et al. 2014; Washietl et al. 2014; Hezroni et al. 2015), with one recent exception (Sarropoulos et al. 2019), have focused on lncRNAs detected in adult organs. We hypothesized that lncRNAs expressed during development may be enriched in functional loci, as suggested by the increasing number of lncRNAs with proposed developmental roles (Rinn et al. 2007; Grote et al. 2013; Sauvageau et al. 2013; Grote and Herrmann 2015). To test this hypothesis, we performed a multidimensional comparative transcriptomics analysis, following lncRNA and protein-coding genes across species, organs, and developmental stages.

We ensured that our transcriptome collection reflects the changes in cellular composition and physiological functions that occur during major organ development, by analyzing cell type-specific gene markers derived from single-cell analyses (Green et al. 2018; Tabula Muris Consortium 2018). We showed that protein-coding gene expression profiles across major organs and developmental stages are well conserved among species, even at large evolutionary distances. Although differences among rodents and chicken are considerable when analyzing the full set of orthologous protein-coding genes (fig. 1), the expression profiles of genes that are known to be implicated in embryonic development and in gene expression regulation processes are highly conserved among species (supplementary fig. 6, Supplementary Material online). Our transcriptome collection thus enables detecting temporal expression patterns shared across divergent species, for key players in developmental regulatory networks. These observations are consistent with findings from a recent publication, which studied protein-coding gene expression patterns during major organ development in amniote species (Cardoso-Moreira et al. 2019). Our transcriptome data set covers a narrower range of species and developmental stages than this comprehensive resource (Cardoso-Moreira et al. 2019), but uniquely includes aged individuals, thus completing the overview of temporal expression patterns. Thus, our work represents an additional resource for evolutionary studies of gene expression.

To our knowledge, together with a recent publication (Sarropoulos et al. 2019), our work is one of the first large-scale lncRNA evolutionary studies that include a temporal dimension, by sampling different developmental stages. Our article and this recent work concur to reveal an enrichment for functional lncRNAs early in development (Sarropoulos et al. 2019). Here, we perform in-depth analyses of expression pattern evolution, short- and long-term sequence evolution for different regions of lncRNAs loci, in conjunction with their expression patterns. We thus bring new insights into the evolution and functionality of lncRNAs.

### Spatio-Temporal lncRNA Expression Patterns

Our first major observation is that lncRNAs are overwhelmingly expressed in the young and aged adult testes (fig. 3), in agreement with previous data (Soumillon et al. 2013). Their relative depletion in embryonic and newborn testes reinforces the association between lncRNA transcription and spermatogenesis, in accord with the hypothesis that the particular chromatin environment during spermatogenesis is a driver for promiscuous, nonfunctional transcription (Kaessmann 2010; Soumillon et al. 2013). Interestingly, we show that lncRNAs are significantly differentially expressed among developmental stages, at least as frequently as protein-coding genes, after correcting for their lower expression levels. However, in contrast with protein-coding genes, the majority of lncRNAs reach their highest expression levels in adult rather than in developing organs (fig. 3). As requirements for tight gene expression control are higher during embryonic development (Ben-Tabou de-Leon and Davidson 2007), an explanation for the relative lncRNA depletion in embryonic and newborn transcriptomes is that transcriptional noise is deleterious and thus more efficiently blocked during the early stages of development. Differences in cellular composition heterogeneity may also be part of the explanation. Expression analyses of cell type-specific markers suggest that adult organ transcriptomes may be a mix of more diverse cell types, including substantial immune cell infiltrates (supplementary fig. 1, Supplementary Material online). A higher cell-type diversity may explain the increased abundance of lncRNAs in young and aged adult organs, especially given that lncRNAs are thought to be cell-type specific (Liu et al. 2016).

We found that lncRNA expression patterns are generally similar between young and aged adult individuals: <50 lncRNAs are significantly DE between these two stages, for most organs (supplementary fig. 9, Supplementary Material online). Moreover, the levels of sequence and expression conservation are globally similar between young and aged adults, for both protein-coding and lncRNA genes (figs. 4 and 7). Overall, our analyses indicate that, with our sampling (supplementary table 1, Supplementary Material online), the physiological processes at work in aged organs are highly similar to those acting in juvenile organs, suggesting that developmental stage sampling should be further extended for in-depth analyses of the aging process.

### Functionally Constrained lncRNAs Are Enriched in Developmental Transcriptomes

Our long-term sequence conservation analyses confirm that lncRNAs are overall under weak, but significant selective constraint (Ponjavic et al. 2007): lncRNA sequence conservation scores are much lower than those of protein-coding genes, but above those of intergenic regions (fig. 4 and supplementary figs.10 and 11, Supplementary Material online). Interestingly, intergenic regions flanking lncRNAs are on an average less conserved than the genomic intergenic average (fig. 4), suggesting that the rapid lncRNA evolution may be a general feature of the genomic regions in which they reside. The underlying mechanisms are unclear, but may reflect a lower density of constrained expression regulatory elements in the vicinity of lncRNAs, or a higher accumulation of lineage-specific transposable elements (Kapusta et al. 2013).

We show that, for those lncRNAs that are expressed above noise levels (TPM≥1) in somatic organs and in the embryonic and newborn developmental stages, there is a higher proportion of evolutionarily constrained loci than in testes-expressed lncRNAs (fig. 4). Strikingly, we find that the level of long-term sequence conservation for lncRNA promoter regions is similar to the one observed for protein-coding promoters, when we analyze genes that are robustly expressed in embryonic brain and kidney. Furthermore, we show that lncRNAs expressed in somatic organs and in the developing testes differ from those expressed in the adult testes not only in terms of overall levels of sequence conservation but also with respect to the regions of the lncRNA loci that are under selective constraint. Thus, for lncRNAs expressed in somatic organs and in the developing testes, there is significantly more evolutionary constraint on promoters and splice sites than on exons, while these patterns are not seen for the bulk of lncRNAs, expressed in adult and aged testes (supplementary fig. 11, Supplementary Material online). We are thus able to modulate previous reports of increased constraint on splicing regulatory regions in mammalian lncRNAs (Schüler et al. 2014; Haerty and Ponting 2015), by showing that this pattern is specific to lncRNAs that are expressed in somatic organs and in the developing testes.

These results are also in agreement with recent findings suggesting that biological function may reside in the presence of additional noncoding regulatory elements at the lncRNA promoter rather than in the production of a specific transcript (Engreitz et al. 2016; Groff et al. 2016). Although the elevated sequence conservation at splicing regulatory signals could indicate that the production of a specific mature lncRNA is required, splicing of lncRNA transcripts was recently proposed to affect the expression of neighboring protein-coding genes (Engreitz et al. 2016). Thus, while there is evidence for increased functionality for lncRNA loci that are detected in developmental transcriptomes or in adult somatic organs, in agreement with a recent report (Sarropoulos et al. 2019), our sequence conservation analyses are compatible with the hypothesis that their biological functions may be carried out in an RNA-independent manner, as exons are under less constraint than promoters or splice sites. Alternatively, their function may be carried out by small conserved elements, such that the sequence conservation on the entire lncRNA exonic sequence is weak (Ulitsky 2016).

### Evolutionary Divergence of Spatio-Temporal Expression Profiles for lncRNAs

We previously showed that lncRNA expression patterns evolve rapidly across species in adult organs (Necsulea et al. 2014). Here, we show that this rapid evolution of lncRNA expression is also true for embryonic and newborn developmental stages. Expression comparisons across species, organs, and developmental stages are dominated by differences between species for lncRNAs (fig. 6), while similarities between organs and developmental stages are predominant for protein-coding genes, even across distantly related species (fig. 1). We assessed the extent of expression conservation by contrasting between-species and within-species expression variations and we showed that lncRNAs have significantly lower levels of conservation than protein-coding genes, for all organs and developmental stages (fig. 7). However, lncRNA expression is more conserved in somatic organs and in early embryonic stages than in the adult testes. Moreover, when orthologous lncRNAs are differentially expressed among developmental stages in both mouse and rat, they generally show parallel profiles of expression variation in both species (fig. 8). This result is in agreement with a recent publication, which showed that temporal patterns of expression variation tend to be evolutionarily conserved for developmentally dynamic lncRNAs (Sarropoulos et al. 2019). We note that these temporal patterns of variation may in fact be caused by spatially restricted lncRNA expression. Previous reports indicated that lncRNA expression may be cell type-specific (Liu et al. 2016). The differentially expressed lncRNAs, shared across mouse and rat, could be specific of cell types that change their relative abundance in whole-organ transcriptomes with developmental time.

Interestingly, when we evaluate expression divergence individually for each orthologous gene pair, correcting for the lower lncRNA expression levels, we find that lncRNAs are not more divergent than protein-coding genes (fig. 8). This observation indicates that much of the between-species differences in lncRNA expression patterns is tightly linked with the low expression levels of lncRNAs. It is not clear however whether this is purely an indication of technical biases, that hamper expression estimation for lowly expressed lncRNAs, or whether the low lncRNA expression levels are a sign that these transcripts are nonfunctional. For cell type-specific lncRNAs, low expression in whole-organ transcriptomes are expected. This question may soon be directly addressed, as single-cell assays become more sensitive and allow investigation of lncRNAs (Liu et al. 2016).

### Candidate Species-Specific lncRNAs

Finally, we analyzed extreme cases of expression divergence between species, where transcription can be robustly detected in one species but not in the other, despite the presence of good sequence conservation. We identify more than a thousand candidate species-specific lncRNAs, in both mouse and rat. Interestingly, we observe that candidate mouse-specific lncRNAs are more frequently transcribed from enhancers than lncRNAs conserved between mouse and rat (supplementary fig. 11, Supplementary Material online). This observation is consistent with previous reports that enhancers and enhancer-associated lncRNAs evolve rapidly (Marques et al. 2013; Villar et al. 2015). Moreover, we show that these lncRNA expression changes do not occur in an isolated manner. When species-specific lncRNA transcription was inferred at protein-coding genes bidirectional promoters, the neighboring protein-coding genes also showed increased expression divergence, compared with genes that are transcribed from conserved lncRNA promoters. We thus confirm that lncRNA turnover is associated with changes in neighboring gene expression (Kutter et al. 2012). Although lncRNAs changes may be directly affecting gene expression, another probable hypothesis is that a common mechanism affects both lncRNAs and protein-coding genes transcribed from bidirectional promoters.

### Conclusions

Our comparative transcriptomics approach confirms that lncRNAs repertoires, sequences, and expression patterns evolve rapidly across species, and shows that accelerated rates of lncRNA evolution are also seen in developmental transcriptomes, albeit less frequently. These observations are consistent with the hypothesis that the majority of lncRNAs (or at least of those detected with sensitive transcriptome sequencing approaches, in particular, in the adult testes) may be nonfunctional. However, we are able to modulate this conclusion, by showing that there are increased levels of functional constraint on lncRNAs expressed during embryonic development, in particular, in the developing brain and kidney. These increased levels of constraint apply to all analyzed aspects of lncRNAs, including sequence conservation for exons, promoter, and splice sites, but also expression pattern conservation. For many of these loci, biological function may be RNA-independent, as the highest levels of selective constraint are observed on promoter regions and on splice signals, rather than on lncRNA exonic sequences. Our results are thus compatible with unconventional, RNA-independent functions for lncRNAs expressed during embryonic development.

## Materials and Methods

### Biological Sample Collection

We collected samples from three species (mouse C57BL/6J strain, rat Wistar strain, and chicken White Leghorn strain), four organs (brain, kidney, liver, and testes) and five developmental stages (including two embryonic stages, newborn, young, and aged adult individuals). We sampled the following stages in the mouse: embryonic day postconception (dpc) 13.5 (E13.5 dpc, hereafter midstage embryo); E17 to E17.5 dpc (late embryo); postnatal days 1–2 (newborn); young adult (8–10 weeks old); aged adult (24 months old). For the rat, we sampled the following stages: E15 dpc (midstage embryo); E18.5 to E19 dpc (late embryo); postnatal days 1–2 (newborn); young adult (8–10 weeks old); aged adult (24 months, with the exception of kidney samples and two of four liver samples, derived from 12-month-old individuals). The embryonic and neonatal developmental stages were selected for maximum comparability based on Carnegie stage criteria (Theiler 1989). For chicken, we collected samples from Hamburger–Hamilton stages 31 and 36 (hereafter termed midstage and late embryo), selected for comparability with the two embryonic stages in mouse and rat (Hamburger and Hamilton 1951). Each sample corresponds to one individual, except for mouse and rat midstage embryonic kidney, for which tissue from several embryos was pooled prior to RNA extraction. For adult and aged organs, multiple tissue pieces from the same individual were pooled and homogenized prior to RNA extraction. For brain dissection, we sampled the cerebral cortex. For mouse and rat, with the exception of the midstage embryonic kidney, individuals were genotyped and males were selected for RNA extraction. Between two and four biological replicates were obtained for each species/organ/stage combination, amounting to 97 samples in total (supplementary table 1, Supplementary Material online).

### RNA-Seq Library Preparation and Sequencing

We performed RNA extractions using RNeasy Plus Mini kit from Qiagen. We assessed RNA quality with the Agilent 2100 Bioanalyzer. RNA integrity numbers (RIN) are available in supplementary table 1, Supplementary Material online; see supplementary methods, Supplementary Material online, for additional RNA integrity analyses. Sequencing libraries were produced with the Illumina TruSeq stranded mRNA protocol with polyA selection, and sequenced as 101 bp single-end reads, at the Genomics Platform of iGE3 and the University of Geneva. Libraries are strand-specific and the sequenced strand is complementary to the RNA molecule.

### Additional RNA-Seq Data

To improve detection power for lowly expressed lncRNAs, we complemented our RNA-seq collection with samples generated with the same technology for Brown Norway rat adult organs (Cortez et al. 2014). We added published data for adult chicken (red jungle fowl strain UCD001) organs (McCarthy et al. 2019), as well as for embryonic chicken (White Leghorn) organs (Ayers et al. 2013; Uebbing et al. 2015). As the data were not comparable with our own in terms of library preparation and animal strains, these samples were only used to increase lncRNA detection sensitivity.

### RNA-Seq Data Processing

We used HISAT2 (Kim et al. 2015) release 2.0.5 to align the RNA-seq data on reference genomes. The genome sequences (assembly versions mm10/GRCm38, rn6/Rnor_6.0 and galGal5/Gallus_gallus-5.0) were downloaded from the Ensembl database (Cunningham et al. 2019). Genome indexes were built using only genome sequence information. To improve detection sensitivity, at the alignment step, we provided known splice junction coordinates extracted from Ensembl. We set the maximum intron length for splice junction detection at 1 Mb. The following command-line arguments were used: –rna-strandness R –known-splicesite-infile=SpliceSites_Ensembl.txt –max-intronlen 1000000 –dta-cufflinks, where SpliceSites_Ensembl.txt corresponds to the exon junction coordinates extracted with hisat2_extract_splice_sites.py. See also supplementary methods, Supplementary Material online, for additional RNA-seq data-quality analyses.

### Transcript Assembly and Filtering

We assembled transcripts for each sample using StringTie (Pertea et al. 2015), release 1.3.5, based on read alignments obtained with HISAT2. We provided genome annotations from Ensembl release 94 as a guide for transcript assembly. We filtered Ensembl annotations to remove transcripts that spanned a genomic length >2.5 Mb. For protein-coding genes, we kept only protein-coding transcripts, discarding isoforms annotated as “retained_intron” and “processed_transcript.” We set the minimum exonic length at 150 bp, the minimum anchor length for splice junctions at 8 bp and the minimum isoform fraction at 0.05. The following StringTie command-line arguments were used: -G Ensembl_annotations.gtf -m 150 -a 8 -f 0.05 –p 8 –rf, where Ensembl_annotations.gtf correspond to the Ensembl annotations filtered as described earlier. We compared the resulting assembled transcripts with Ensembl annotations and we discarded read-through transcripts, overlapping with multiple multiexonic Ensembl-annotated genes. For strand-specific samples, we discarded transcripts for which the ratio of sense to antisense unique read coverage was <0.01. We discarded multiexonic transcripts that were not supported by splice junctions with correctly assigned strands. The filtered transcripts obtained for each sample were assembled into a single data set *per* species using the merge option in StringTie. For increased sensitivity, we removed the minimum FPKM and TPM thresholds, but required a minimum isoform fraction of 0.05 for transcript inclusion. The following StringTie command-line arguments were used: stringtie -v –merge -G Ensembl_annotations.gtf -m 150 -a 8 -p 8 -F 0 -T 0 -f 0.05. We constructed a combined annotation data set, starting with Ensembl annotations, to which we added newly assembled transcripts that had no exonic overlap with Ensembl genes. We also included newly annotated isoforms for known genes if they had exonic overlap with exactly one Ensembl gene, thus discarding potential read-through transcripts or gene fusions.

### Protein-Coding Potential of Assembled Transcripts

To determine whether the newly assembled transcripts were protein-coding or noncoding, we mainly relied on the codon substitution frequency (CSF) score (Lin et al. 2007). As in a previous publication (Necsulea et al. 2014), we scanned whole-genome alignments and computed CSF scores in 75 bp sliding windows moving with a 3-bp step. We used precomputed alignments downloaded from the UCSC Genome Browser (Casper et al. 2018), including the alignment between the mouse genome and 59 other vertebrates (for mouse classification), between the human genome and 99 other vertebrates (for rat and chicken classification) and between the rat genome and 19 other vertebrates (for rat classification). For each window, we computed the score in each of the six possible reading frames and extracted the maximum score for each strand. We considered that transcripts are protein-coding if they overlapped with positive CSF scores on at least 150 bp. As positive CSF scores may also appear on the antisense strand of protein-coding regions due to the partial strand-symmetry of the genetic code, in this analysis, we considered only exonic regions that did not overlap with other genes. In addition, we searched for sequence similarity between assembled transcripts and known protein sequences from the SwissProt 2017_04 (The UniProt Consortium 2017) and Pfam 31.0 (El-Gebali et al. 2019) databases. We kept only SwissProt entries with confidence scores 1, 2, or 3 and we used the Pfam-A curated section of Pfam. We searched for sequence similarity using the BlastX utility in the BLAST+ 2.8.1 package (Altschul et al. 1990; Camacho et al. 2009), keeping hits with maximum e-value 1e^−3^ and minimum protein sequence identity 40%, on repeat-masked cDNA sequences. We considered that transcripts were protein-coding if they overlapped with BlastX hits over at least 150 bp. Genes were said to be protein-coding if at least one of their isoforms was classified as protein-coding, based on either the CSF score or on sequence similarity with known proteins.

### Long Noncoding RNA Selection

To construct a reliable lncRNA data set, we selected newly annotated genes classified as noncoding based on both the CSF score and on sequence similarity with known proteins and protein domains, as well as Ensembl-annotated genes with noncoding biotypes (“lincRNA,” “processed_transcript,” “antisense,” “TEC,” “macro_lncRNA,” “bidirectional_promoter_lncRNA,” “sense_intronic”). For newly detected genes, we applied several additional filters: we required a minimum exonic length (corresponding to the union of all annotated isoforms) of at least 200 bp for multiexonic loci and of at least 500 bp for mono-exonic loci; we eliminated genes that overlapped for >5% of their exonic length with unmappable regions; we kept only loci that were classified as intergenic and at least 5 kb away from Ensembl-annotated protein-coding genes on the same strand; for multiexonic loci, we required that all splice junctions be supported by reads with correct strand assignment (cf. above). For both de novo and Ensembl annotations, we removed transcribed loci that overlapped on at least 50% of their length with retrotransposed gene copies, annotated by the UCSC Genome Browser and from a previous publication (Carelli et al. 2016); we discarded loci that overlapped with UCSC-annotated tRNA genes and with RNA-type elements from RepeatMasker (Smit et al. 2003) on at least 25% of their length. We kept loci supported by at least ten uniquely mapped RNA-seq reads and for which a ratio of sense to antisense transcription of at least 1% was observed in at least one sample. Although the fraction of reads stemming from the wrong strand due to errors in library preparations is very low in our samples (supplementary table 1, Supplementary Material online), loci situated on the antisense strand of highly expressed genes can have unreliable expression estimates. Thus, for loci that had sense/antisense exonic overlap with other genes, we computed expression levels either on complete gene annotations, or only on exonic regions that had no overlap with other genes, and computed Spearman’s correlation coefficient between the two expression estimates, across all samples. We discarded loci for which the correlation coefficient was <0.9. Full gene annotations and lncRNA selection criteria are provided in supplementary data set 1, Supplementary Material online.

### Gene Expression Estimation

We computed the number of uniquely mapping reads unambiguously attributed to each gene using the Rsubread package in R (Liao et al. 2019), discarding reads that overlapped with multiple genes. We also estimated read counts and TPM values *per* gene using Kallisto (Bray et al. 2016). To approach absolute expression levels estimates, for better comparisons across samples, we further normalized TPM values using a scaling approach (Brawand et al. 2011). Briefly, we ranked the genes in each sample according to their TPM values, we computed the variance of the ranks across all samples for each gene, and we identified the 100 least-varying genes, found within the interquartile range (25–75%) in terms of average expression levels across samples. We derived normalization coefficients for each sample such that the median of the 100 least-varying genes be identical across samples. We then used these coefficients to normalize TPM values for each sample. We excluded mitochondrial genes from expression estimations and analyses, as these genes are highly expressed and can be variable across samples. For differential expression analyses, we used *per*-gene unique read counts computed with Rsubread. For all downstream analyses, we used normalized TPM values. When indicated, we transformed TPM values with the following formula: *x*->log2(*x* + 1). Gene expression data are available in supplementary data set 2, Supplementary Material online.

### Differential Expression Analyses

We used the DESeq2 (Love et al. 2014) package release 1.22.2 in R release 3.5.0 (R Core Team 2018) to test for differential expression across developmental stages, separately for each organ and species. We analyzed both protein-coding genes and lncRNAs, selected according to the criteria described earlier. We first performed a global differential expression analysis, using the likelihood ratio test to contrast a model including an effect of the developmental stage against the null hypothesis of homogeneous expression across all developmental stages. This analysis was performed on all protein-coding and lncRNA genes for each species, as well as on 1-to-1 orthologs for mouse and rat. In addition, we down-sampled the numbers of reads assigned to protein-coding genes to obtain identical average numbers of reads for protein-coding genes and lncRNAs. The resampled read counts were directly proportional to the original counts for each protein-coding gene. We also contrasted consecutive developmental stages, for each species and organ, using the Wald test implemented in DESeq2. Differential expression results are available in supplementary data set 4, Supplementary Material online.

### Homologous lncRNA Family Prediction

We used existing whole-genome alignments as a guide to predict homologous lncRNAs across species, as previously proposed (Washietl et al. 2014). We first constructed for each gene the union of its exon coordinates across all isoforms, hereafter termed “exon blocks.” We projected exon block coordinates between pairs of species using the liftOver utility and whole-genome alignments generated with BlastZ (http://www.bx.psu.edu/miller_lab/; last accessed September 24, 2019), available through the UCSC Genome Browser (Casper et al. 2018). To increase detection sensitivity, for the initial liftOver projection, we required only that 10% of the reference bases remap on the target genome. Projections were then filtered, retaining only cases where the size ratio between the projected and the reference region was between 0.33 and 3 for mouse and rat (0.2 and 5 for comparisons involving chicken). To exclude recent lineage-specific duplications, regions with ambiguous or split liftOver projections were discarded. For genes where multiple exon blocks could be projected across species, we defined the consensus chromosome and strand in the target genome and discarded projected exon blocks that did not match this consensus. We then evaluated the order of the projected exon blocks on the target genes, to identify potential internal rearrangements. If internal rearrangements were due to the position of a single projected exon block, the conflicting exon block was discarded; otherwise, the entire projected gene was eliminated. As the projected reference gene coordinates could overlap with multiple genes in the target genome, we constructed gene clusters based on the overlap between projected exon block coordinates and target annotations, using a single-link clustering approach. We then realigned entire genomic loci for each pair of reference-target genes found within a cluster, using lastz (http://www.bx.psu.edu/miller_lab/) and the threaded blockset aligner TBA (Blanchette et al. 2004). Using this alignment, we computed the percentage of exonic sequences aligned without gaps and the percentage of identical exonic sequence, for each pair of reference-target genes. We then extracted the best hit in the target genome for each gene in the reference genome based on the percentage of identical exonic sequence, requiring that the ratio between the maximum percent identity and the percent identity of the second-best hit be >1.1. Reciprocal best hits were considered to be 1-to-1 orthologous loci between pairs of species. For analyses across all three species, we constructed clusters of reciprocal best hits from pairwise species comparisons, using a single-link clustering approach. Resulting clusters with more than one representative *per* species were discarded. The results of the homology prediction pipeline, sequence alignment statistics and Ensembl orthology relationships for protein-coding genes are available in supplementary data set 5, Supplementary Material online.

## Data Availability

The RNA-seq data were submitted to the NCBI Gene Expression Omnibus (GEO), under accession number GSE108348. Supplementary data sets, Supplementary Material online, containing additional processed data are available at the address: ftp://pbil.univ-lyon1.fr/pub/datasets/Darbellay_LncEvoDevo. The scripts used to analyze the data are available through a GitHub repository: https://github.com/anecsulea/LncEvoDevo. See also supplementary methods, Supplementary Material online.

## Supplementary Material

msz212_Supplementary_Data
