# Supplementary material for: Comparative Transcriptomics Analyses across Species, Organs, and Developmental Stages Reveal Functionally Constrained lncRNAs
Source: Mol Biol Evol. 2019 Sep 20;37(1):240–59. doi: 10.1093/molbev/msz212 (PMC6984365; doi:10.1093/molbev/msz212)

Supplementary Methods 3

RNA-seq data quality analyses 3

RNA integrity analysis 3

RNA-seq read downsampling to homogenize read coverage 3

LncRNA and protein-coding gene promoter types 3

Gene expression specificity 4

Gene markers for organs and developmental stages 4

Gene ontology enrichment 4

Definition of developmental transcription factors 5

Principal component analyses of gene expression patterns 5

Sequence evolution 5

Gene expression evolution 6

Between-species comparison of temporal expression patterns 6

Transcription factor binding site enrichment 7

Candidate species-specific lncRNAs 7

Statistical analyses and graphical representations 7

Supplementary Figure Legends. 8

Supplementary Figure 1. Expression patterns of cell-type specific markers in mouse, rat and chicken samples. 8

Supplementary Figure 2. Genes with narrow expression distribution across organs and developmental stages, with maximum expression observed in brain. 8

Supplementary Figure 3. Genes with narrow expression distribution across organs and developmental stages, with maximum expression observed in kidney. 8

Supplementary Figure 4. Genes with narrow expression distribution across organs and developmental stages, with maximum expression observed in liver. 9

Supplementary Figure 5. Genes with narrow expression distribution across organs and developmental stages, with maximum expression observed in testes. 9

Supplementary Figure 6. Global patterns of gene expression variation across species, organs and developmental stages. 10

Supplementary 7. Expression level distribution for different classes of lncRNAs, for mouse, rat and chicken. 10

Supplementary Figure 8. Patterns of differential expression among developmental stages for protein-coding genes and lncRNAs. 11

Supplementary Figure 9. Number of differentially expressed genes between consecutive developmental stages. 11
Supplementary Figure 10. Long-term sequence conservation for lncRNA loci. 11
Supplementary Figure 11. Estimates of long-term sequence conservation scores for different regions of lncRNA loci. 12
Supplementary Figure 12. Gene structure conservation for orthologous lncRNAs. 12
Supplementary Figure 13. Conservation of developmental expression profiles between mouse and rat, for orthologous protein-coding genes. 13
Supplementary Figure 14. Expression pattern divergence between mouse and rat. 13
Supplementary Figure 15. Candidate species-specific lncRNAs. 14
Supplementary Figure 16. Genomic and expression characteristics of candidate species-specific lncRNAs. 14

Supplementary Table List 15

Supplementary Dataset List 16

References 17

Supplementary Figures 18

Supplementary Methods

RNA-seq data quality analyses

To verify the strandedness of the RNA-seq data, we analyzed spliced reads that spanned introns with canonical (GT-AG or GC-AG) splice sites and compared the strand inferred based on the splice site with the one assigned based on the library preparation protocol (Supplementary Table 1). To estimate the mappability of each genomic region, we generated error-free artificial RNA-seq reads (single-end, 101 bp long, consecutive read starts spaced by 5 bp) from the genome sequence and realigned them to the genome with the same HISAT2 parameters. Regions for which the corresponding reads could be aligned unambiguously were considered mappable; remaining regions were said to be unmappable.

RNA integrity analysis

We evaluated RNA quality prior to library preparation with the Agilent 2100 Bioanalyzer tool. Most samples had RIN values above 9 and only 4 out of 97 sequenced libraries had RIN values comprised between 7.5 and 8 (Supplementary Table 1). To further assess the quality of the sequenced libraries, we analyzed the pattern of read coverage variation along genes. For polyA-selected libraries, RNA degradation is reflected in the presence of higher read coverage in the 3' region of the mRNAs compared to the 5' region. We divided the exonic sequences of Ensembl-annotated mRNAs into 20 equal-size windows, and computed the *per*-base unique read coverage in each window, averaged across all Ensembl-annotated protein-coding genes. We used only mRNAs that were at least 400 bp long. As expected given that transcript ends are difficult to sample with RNA-seq, we observed that the first and last windows also show decreased read coverage compared to the rest of the isoform. We thus excluded the first 4 and the last 3 window before fitting a linear regression between the average read coverage per window and the window position in the gene. We used the slope of this linear regression as an indicator of RNA degradation (3' bias column in Supplementary Table 1).

RNA-seq read downsampling to homogenize read coverage

We observed that some organ / developmental stage combinations express more protein-coding genes and lncRNAs than others. To verify that this observation is not affected by unequal RNA-seq read coverage, we homogenized the read coverage among organ / developmental stage combinations, by randomly resampling the same number of uniquely mapped reads. We resampled 60 million reads for mouse and rat and 50 million reads for chicken, combining biological replicates for each organ / developmental stage combination.

LncRNA and protein-coding gene promoter types

We assessed the proximity between the transcription start sites (TSS) of lncRNA loci and other genes TSS, to determine whether lncRNA promoters are unidirectional or bidirectional. We said that lncRNAs or protein-coding genes have bidirectional promoters if at least one of their annotated TSS is found

within 1 kb of a different gene TSS. We also analyzed the proximity between promoter regions and Encode-annotated enhancers, for mouse (Shen et al. 2012). We combined all enhancer coordinates across all tissues, and converted their coordinates between mm9 and mm10 genome assemblies with liftOver. This resulted in 400,017 predicted enhancer regions.

Gene expression specificity

We used the previously proposed tissue specificity index (Liao et al. 2006) to measure gene expression specificity across organs and developmental stages, provided by the formula: tau = sum $(1 - r_i)/(n-1)$, where r_i represents the ratio between the expression level in sample i and the maximum expression level across samples, and n represents the total number of samples. We computed this index on normalized TPM values, averaged across all replicates for a given species / organ / developmental stage combination (Supplementary Dataset 3). The average expression values across biological replicates were also used to evaluate the sample (organ / developmental stage combination) in which maximum expression is reached, for each gene. The organ and developmental stage in which maximum expression is reached (plotted in Figures 3, 5, 8 and Supplementary Figure 13) is derived from this computation. Gene expression statistics are available in Supplementary Dataset 3 online.

Gene markers for organs and developmental stages

To validate our comparative transcriptome collection and to scrutinize the cell composition changes that occur during major organ development, we analyzed the expression patterns of cell-type specific genes. We extracted proposed cell-type specific markers from mouse single-cell RNA-seq studies (Tabula Muris Consortium 2018; Green et al. 2018), and filtered them to keep genes that are associated with a single cell type, with orthologues in rat and/or chicken (Supplementary Table 5). In addition, we identified genes that could be used as putative markers for organs and developmental stages, based on our transcriptome collection. To do this, we extracted protein-coding genes that had high expression specificity indexes (tau >= 0.85) in both mouse and rat, and for which the organ/stage in which maximum expression is observed is the same for both species. We selected genes that had an expression level (TPM) above 2 in at least one sample, for each species. For this analysis, we combined the young and aged adult samples. The predicted cell-type specific markers and gene ontology enrichment analyses (comparing these gene lists with the entire set of protein-coding genes that have 1-to-1 orthologues in mouse and rat) are available in Supplementary Dataset 3 online.

Gene ontology enrichment

We extracted gene ontology annotations for mouse, rat and chicken from the Ensembl 94 database, using BioMart (Smedley et al. 2015). We evaluated the enrichment of gene ontology categories between a target gene set (e.g., genes that are differentially expressed between two developmental stages, in a given organ) and a background gene set (e.g., all genes expressed in that organ), using the

hypergeometric test in R. We performed the gene ontology enrichment for the “biological process” gene ontology category, and set the false discovery rate (FDR) threshold at 0.1.

Definition of developmental transcription factors

For the developmental transcription factor analysis presented in Supplementary Figure 6, we selected genes associated with at least one of the following categories: multicellular organism development, system development, embryonic organ development, animal organ development or pattern specification process. We further selected from the resulting list those genes associated with at least one of the following categories: regulation of gene expression, gene expression, positive regulation of gene expression, negative regulation of gene expression, regulation of transcription by RNA polymerase II, regulation of transcription, DNA-templated.

Principal component analyses of gene expression patterns

Principal component analyses were performed on log2-transformed TPM values, using functions in the ade4 library in R (Dray and Dufour 2007). The input data was a matrix of gene expression profiles across genes and samples, centered and scaled prior to analysis. We observed that sample coordinates on the axis 1 (for brain and testis) and 2 (for kidney and liver) of the principal component analysis correlate with the developmental stage from which the samples were derived. To control for potential confounding factors, we verified whether the coordinates on PC1 and PC2 were correlated with RNA integrity values (RIN), 3’ read coverage bias, or library size. As the coordinates on PC1 and PC2 also appeared to be correlated with the 3’ read coverage bias, we fit a linear regression between the coordinates on the axes and the 3’ read coverage bias, and verified that the residuals of the linear regression correlate with the developmental stage from which the samples were derived with a Kruskal-Wallis test (Supplementary Figure 6).

Sequence evolution

We evaluated long-term evolutionary sequence conservation based on PhastCons (Siepel et al. 2005) scores, computed for the mouse genome using either a placental mammal multiple species alignment, available from the UCSC Genome Browser (Casper et al. 2018). We attributed a value of 0 to all genomic positions that are not present in the whole-genome alignment used for PhastCons computations. We computed average PhastCons scores on exonic sequences (excluding exonic regions overlapping with other genes), promoter regions (defined as 400 bp immediately upstream of the transcription start site, masking any exonic regions that overlapped in these regions) and splice sites (defined as the first two and last two bases of each intron). For genes with multiple promoters, we computed the average score across all promoters. In addition, we evaluated the amount of sequence conservation in intergenic regions. To define intergenic regions, we extended the coordinates of all transcribed loci (either Ensembl-annotated or identified with StringTie, coding or

non-coding) by 5 kb on each side, and we extracted the complementary genomic regions. We analyzed two sets of intergenic regions: all intergenic regions that were at least 1kb long, or intergenic regions that flanked candidate lncRNAs. For this latter subset, we extracted 2.5 kb long segments situated exactly 5 kb away from lncRNA loci and we selected those segments that were at least 5 kb away from other transcribed loci. This resulted in 8496 regions. In the genome-wide set there were 22628 intergenic regions. Sequence conservation statistics for lncRNAs are provided in Supplementary Table 5; all sequence conservation data are provided in Supplementary Dataset 5.

Gene expression evolution

We evaluated expression pattern conservation at two scales: first globally, for an entire gene category, and second on a gene-by-gene basis. Both analyses were performed by comparing mouse and rat expression patterns, for 1-to-1 orthologous genes.

First, we measured gene expression conservation for protein-coding genes and lncRNAs as a class. To do this, for each organ/developmental stage, we computed average expression levels (normalized TPM) across biological replicates, for each species. across all orthologous gene pairs. We also computed the correlation between individuals within the same species; for organ/stages with more than two biological replicates we computed the average across all possible pairs of individuals. We then computed the ratio of the between-species correlation coefficient to the average within-species correlation coefficient. Spearman's rank correlation coefficients were used in all cases. We obtained confidence intervals for expression conservation measures through a bootstrap procedure, resampling 100 times the same number of genes with replacement.

Second, we computed Euclidean distances between relative expression profiles, for each orthologous gene pair. The relative expression profiles were derived from normalized TPM values *per* organ/developmental stage, averaged across biological replicates within each species, divided by the sum of all average TPM values. We observed that this measure of expression divergence correlates negatively with the average expression level (Figure 9). To correct for this effect, we fitted a linear regression between the expression divergence measure and the average expression level, and extracted the residuals of the linear regression. We computed the contribution of each organ / developmental stage combination to the expression divergence measure through the ratio of the squared difference between species for that sample to the squared Euclidean distance. The sum of the individual contributions of all organ / developmental stage combinations is thus equal to 1.

Between-species comparison of temporal expression patterns

To determine whether broad patterns of gene expression variation among developmental stages are conserved during evolution, we analyzed 1-to-1 orthologous lncRNAs and protein-coding genes that were significantly DE (FDR < 0.01) in both mouse and rat, when testing for a global effect of the

developmental stage factor, for each organ independently (cf. Differential expression analyses subsection above). For these genes, we constructed relative expression profiles by averaging TPM values across biological replicates for each developmental stage and dividing them by the maximum value, separately for each organ and species. The resulting relative expression profiles were combined across species and clustered with the K-means algorithm (Hartigan and Wong 1979) in R (Figure 8, Supplementary Figure 13, Supplementary Dataset 4). We set the numbers of clusters at 5 for somatic organs and at 4 for the testes, corresponding to the number of developmental stages sampled. For protein-coding gene clusters, we tested for gene ontology enrichment by comparing the list of protein-coding genes in each cluster with the full list of DE protein-coding genes, with a hypergeometric test. The results of the differential expression analyses, K-means clustering and gene ontology enrichment are in Supplementary Dataset 4 online.

Transcription factor binding site enrichment

We used Homer (Heinz et al. 2010) to evaluate the enrichment of transcription factor binding sites in the promoter regions of protein-coding genes and lncRNAs in the previously defined K-means clusters (see above). We defined promoters as 400 bp regions upstream of the transcription start site. For genes with multiple transcription start sites, only the most distal one was used. We contrasted the set of protein-coding genes or lncRNAs in a given K-means cluster with the full set of genes (from the same category) expressed (TPM>0) in that organ. Enrichment analyses were performed separately for mouse and rat. Results are provided in Supplementary Table 8.

Candidate species-specific lncRNAs

To analyze extreme cases of lncRNA expression changes between species, we predicted candidate species-specific lncRNAs, for which the exonic sequence is well aligned between the two species, but transcription could only be detected in one of the two species. We selected loci for which 100% of the exonic sequence could be projected in the other species and passed all filtering steps that discard local rearrangements etc. We asked that the lncRNA be supported by a minimum of 100 reads in the reference species and that no RNA-seq reads could be aligned in the corresponding region in the target species. Similar results were obtained with a more stringent threshold of 250 reads (Supplementary Dataset 8).

Statistical analyses and graphical representations

All statistical analyses and graphical representations were done with R (R Core Team 2018), version 3.5.0. We performed principal component analyses using the ade4 library (Dray and Dufour 2007) and hierarchical clustering of gene expression matrices using the hclust function in the stats package in R, on distance matrices, where the distance between two samples is defined by 1-rho and rho is Spearman's correlation coefficient. For all analyses involving multiple statistical tests, false discovery

rates were computed with the Benjamini-Hochberg procedure (Benjamini and Hochberg 1995). 95% confidence intervals for median values of distributions were computed with the following formula: median +/- 1.57 x IQR/sqrt(N), where IQR is the inter-quartile range, sqrt denotes the square root and N the number of points.

Supplementary Figure Legends.

Supplementary Figure 1. Expression patterns of cell-type specific markers in mouse, rat and chicken samples.

Expression of cell type-specific markers derived from single-cell experiments (full list provided in Supplementary Table 3), in our mouse and rat RNA-seq samples, averaged across biological replicates. The heatmap represents centered and scaled log2-transformed TPM levels (z-score). Developmental stages are indicated by numeric labels, 1 to 5. Species are color-coded, shown below the heatmap.

Supplementary Figure 2. Genes with narrow expression distribution across organs and developmental stages, with maximum expression observed in brain.

A. Expression pattern (normalized TPM expression levels) across organs, species and developmental stages for *Fezf1*, which is predominantly expressed in brain mid-stage embryo, in both mouse and rat.

B. Gene ontology enrichment for genes with narrow expression distribution (organ/stage specificity index >= 0.85) and maximum expression reached in brain mid-stage embryo, in both mouse and rat.

C. Same as A, for *Neurod6*, which is predominantly expressed in brain late embryo.

D. Same as B, for genes with maximum expression in brain late embryo.

E. Same as A, for *Hs3st5*, which is predominantly expressed in brain newborn.

F. Same as B, for genes with maximum expression in brain newborn.

G. Same as A, for *Mobp*, which is predominantly expressed in brain young or aged adult.

H. Same as B, for genes with maximum expression in brain young or aged adult.

Full list of genes is provided in Supplementary Table 4.

Supplementary Figure 3. Genes with narrow expression distribution across organs and developmental stages, with maximum expression observed in kidney.

A. Expression pattern (TPM expression levels) across organs, species and developmental stages for *Hmga2*, which is predominantly expressed in kidney mid-stage embryo, in both mouse and rat.

B. Gene ontology enrichment for genes with narrow expression distribution (organ/stage specificity index >= 0.85) and maximum expression reached in kidney mid-stage embryo, in both mouse and rat.

C. Same as A, for *Foxl1*, which is predominantly expressed in kidney late embryo.

D. Same as A, for *Atp12a,* which is predominantly expressed in kidney newborn.

E. Same as A, for *Dnase1*, which is predominantly expressed in kidney young or aged adult.

F. Same as B, for genes with maximum expression in kidney young or aged adult.

Full list of genes is provided in Supplementary Table 4.

Supplementary Figure 4. Genes with narrow expression distribution across organs and developmental stages, with maximum expression observed in liver.

A. Expression pattern (TPM expression levels) across organs, species and developmental stages for *Hbb-bh1*, which is predominantly expressed in liver mid-stage embryo, in both mouse and rat.

B. Gene ontology enrichment for genes with narrow expression distribution (organ/stage specificity index >= 0.85) and maximum expression reached in liver mid-stage embryo, in both mouse and rat.

C. Same as A, for *Rbp2*, which is predominantly expressed in liver late embryo.

D. Same as B, for genes with maximum expression in liver late embryo.

E. Same as A, for *Igfbp1*, which is predominantly expressed in liver newborn.

F. Same as B, for genes with maximum expression in liver newborn.

G. Same as A, for *Sdr9c7*, which is predominantly expressed in liver young or aged adult.

H. Same as B, for genes with maximum expression in liver young or aged adult.

Full list of genes is provided in Supplementary Table 4.

Supplementary Figure 5. Genes with narrow expression distribution across organs and developmental stages, with maximum expression observed in testes.

A. Expression pattern (TPM expression levels) across organs, species and developmental stages for *Piwil4*, which is predominantly expressed in testes late embryo, in both mouse and rat.

B. Gene ontology enrichment for genes with narrow expression distribution (organ/stage specificity index >= 0.85) and maximum expression reached in testes late embryo, in both mouse and rat.

C. Same as A, for *Aqp12*, which is predominantly expressed in testes newborn.

D. Same as B, for genes with maximum expression in testes newborn.

E. Same as A, for *Defb33*, which is predominantly expressed in testes young or aged adult.

F. Same as B, for genes with maximum expression in testes young or aged adult.

Full list of genes is provided in Supplementary Table 4.

Supplementary Figure 6. Global patterns of gene expression variation across species, organs and developmental stages.

A. Relationship between developmental stages (numbered 1 to 5) and the coordinates on the PCA axes 1 and 2, for each organ separately. For brain and testes, we show the coordinates on PC1, for kidney and liver, we show the coordinates on PC2, which are correlated with developmental stages. The PCA was performed on the full set of 10,363 protein-coding genes with orthologues in mouse, rat and chicken (Supplementary Methods, Figure 1). P-values for Kruskal-Wallis tests, verifying if coordinates on PCA axes are associated with the developmental stages from which samples are derived, are shown above the plots.

B. Relationship between developmental stages (numbered 1 to 5) and the coordinates on the PCA axes 1 and 2, after correcting for the 3' read coverage bias. The Y axis represents the residuals of a linear regression between the samples coordinates on the PCA axes and the 3' read coverage bias (Supplementary Methods). P-values for Kruskal-Wallis tests are shown above the plots.

C. First factorial map of a principal component analysis, performed on log2-transformed TPM values, for 289 protein-coding genes with orthologues in mouse, rat and chicken, associated with organism development and regulation of gene expression in mouse gene ontology (Materials and methods). Colors represent different organs and developmental stages, point shapes represent different species.

D. Hierarchical clustering, performed on a distance matrix derived from Spearman correlations between pairs of samples, for 289 protein-coding genes associated with organism development and regulation of gene expression, with 1-to-1 orthologues in mouse, rat and chicken. Organ and developmental stages are color-coded, shown below the heatmap. Species of origin is color-coded, shown on the right. Sample clustering is shown on the left.

Supplementary 7. Expression level distribution for different classes of lncRNAs, for mouse, rat and chicken.

A. Distribution of the maximum expression level (log2-transformed TPM), for mouse protein-coding genes and lncRNAs.

B. Distribution of the maximum expression level, for different classes of lncRNAs, in the mouse. From left to right: all lncRNAs, spliced (multi-exonic) lncRNAs, unspliced (mono-exonic) lncRNAs, lncRNAs with bidirectional promoters shared with protein-coding genes, lncRNAs with bidirectional promoters shared with other types of genes, lncRNA with unidirectional promoters, antisense lncRNAs (that have exonic or intronic overlap with protein-coding genes on the opposite strand), intergenic lncRNAs (that have no overlap with protein-coding genes on the opposite strand), lncRNAs that have an Encode-

annotated enhancer within 1kb of their transcription start site, lncRNAs that are further away from Encode-annotated enhancers.

C. Same as A, for the rat.

D. Same as B, for the rat. For this species, we did not analyze the proximity between lncRNA promoters and enhancers, for lack of data.

E. Same as A, for the chicken.

F. Same as B, for the chicken. For this species, we did not analyze the proximity between lncRNA promoters and enhancers, for lack of data.

Supplementary Figure 8. Patterns of differential expression among developmental stages for protein-coding genes and lncRNAs.

A. Distribution of the relative expression change, defined as the difference between the maximum and the minimum expression level across developmental stages, normalized by the maximum expression level, for mouse and rat protein-coding genes and lncRNAs that are significantly differentially expressed (FDR<0.01) among developmental stages. Higher values indicate higher fold expression changes.

B. Distribution of the developmental stage in which the maximum expression is observed, for rat protein-coding genes and lncRNAs that are significantly differentially expressed (FDR<0.01) among developmental stages.

A,B. Differential expression analyses are performed separately for each organ.

Supplementary Figure 9. Number of differentially expressed genes between consecutive developmental stages.

Numbers of significantly up-regulated and down-regulated protein-coding genes and lncRNAs (FDR<0.01). Differential expression tests are performed separately for each organ and species, comparing consecutive developmental stages.

Supplementary Figure 10. Long-term sequence conservation for lncRNA loci.

A. Distribution of the sequence conservation score **(**PhastCons), for protein-coding and lncRNAs exonic regions, as well as for intergenic regions, in the mouse (Supplementary Methods). We used precomputed PhastCons score for placental mammals, downloaded from the UCSC Genome Browser. Exonic regions that overlap with exons from other genes were masked. Boxplot notches represent 95% confidence intervals of the medians. Numbers of analyzed genes are shown below the plot. Red:

protein-coding genes; blue: lncRNA loci; gray: all intergenic regions or intergenic regions flanking lncRNA loci Supplementary Methods).

B. Distribution of the sequence conservation score, for promoter regions (400 bp upstream of transcription start sites) of mouse protein-coding genes and lncRNAs. Exonic sequences were masked in promoter regions. Genes are divided in different classes depending on their promoter type: unidirectional, bidirectional shared with protein-coding genes, bidirectional shared with non-coding genes, overlap with Encode-annotated enhancers. The gray boxplots represent all intergenic regions, genome-wide, or intergenic regions flanking lncRNA loci (Supplementary Methods).

C. Distribution of the sequence conservation score, for splice sites (first and last two bases of each intron). The gray boxplots represent all intergenic regions, genome-wide, or intergenic regions flanking lncRNA loci (Supplementary Methods).

Supplementary Figure 11. Estimates of long-term sequence conservation scores for different regions of lncRNA loci.

A. Distribution of the promoter sequence conservation score (PhastCons) for lncRNAs that have unidirectional promoters (no other transcription start site within 1kb of the start of the locus), and which are significantly expressed (TPM>=1) in each organ and developmental stage. Precomputed PhastCons score for placental mammals were downloaded from the UCSC Genome Browser. Exonic regions that overlap with other genes were masked. Dots represent median values, vertical bars represent 95% confidence intervals. The gray dots and vertical bars represent the median value and 95% confidence interval for all intergenic regions, genome-wide, or for intergenic regions flanking lncRNA loci (Supplementary Methods).

B. Same as A, for lncRNAs that have bidirectional promoters shared with protein-coding genes.

C. Same as A, for lncRNAs that have bidirectional promoters shared with other types of genes (non-coding).

D. Distribution of the difference between the exonic sequence conservation (PhastCons) score and the promoter score, for mouse lncRNAs that are significantly expressed (TPM>=1) in each organ and developmental stage.

E. Same as D, for the difference between exonic and splice site sequence conservation score.

Supplementary Figure 12. Gene structure conservation for orthologous lncRNAs.

A. Distribution of the percentage of exonic sequence aligned without gaps, with respect to the maximum exonic length, for each pair of orthologous genes, between mouse and rat. Red: protein-

coding genes, blue: lncRNAs. Sequence alignment was performed with the Threaded Blockset Aligner (Blanchette et al. 2004).

B. Distribution of the percentage of identical exonic sequence, with respect to the exonic sequence length aligned without gaps, for each pair of orthologous genes. Red: protein-coding genes, blue: lncRNAs.

C. Distribution of the relative difference of the number of exons, defined as the absolute difference of the number of exons of the two species, divided by the maximum number of exons across species, for each pair of orthologous genes. Red: protein-coding genes, blue: lncRNAs.

Supplementary Figure 13. Conservation of developmental expression profiles between mouse and rat, for orthologous protein-coding genes.

A. Comparison of the developmental stage in which maximum expression is observed, for orthologous protein-coding genes that are significantly differentially expressed (FDR<0.01) among developmental stages, for both mouse and rat. Genes are divided based on the developmental stage where maximum expression is observed in mouse organs (X-axis). The Y axis represents the percentage of orthologous genes that reach maximum expression in each developmental stage, in the rat. Numbers of analyzed genes are provided below the plot.

B. Expression profiles of orthologous protein-coding genes that are significantly differentially expressed (FDR<0.01) among developmental stages, for both mouse and rat, in the brain. TPM values were averaged across replicates and normalized by dividing by the maximum, for each species. The resulting relative expression profiles were combined across species and clustered with the K-means algorithm. The average profiles of the genes belonging to each cluster are shown. Gray lines represent profiles of individual genes from a cluster. Numbers of genes in each cluster are shown in the plot.

C. Same as B, for the kidney.

D. Same as B, for the liver.

E. Same as B, for the testes. For this organ, we searched for only 4 clusters with the K-means algorithm. The first and second cluster (1,182 and 1,509 genes, respectively, corresponding to 25% of all DE genes in the testes), are representative of genes with different temporal expression profiles in mouse and rat.

Supplementary Figure 14. Expression pattern divergence between mouse and rat.

A. Distribution of raw expression divergence values for different classes of protein-coding genes and lncRNAs, depending on their promoter type in mouse: unidirectional, bidirectional shared with

protein-coding genes, bidirectional shared with non-coding genes, overlap with Encode-annotated enhancers.

B. Same as A, for the residual expression divergence values, after correction for the average expression levels.

C. Examples of expression profiles in mouse and rat, for the top 2 most-divergent protein-coding and lncRNA genes.

<u>Supplementary Figure 15. Candidate species-specific lncRNAs.</u>

Genomic localization and RNA-seq read coverage of a candidate mouse-specific lncRNA, situated downstream of the *Fzd4* gene. RNA-seq data is shown for young and aged adult kidney.

<u>Supplementary Figure 16. Genomic and expression characteristics of candidate species-specific lncRNAs.</u>

A. Percentage of mouse lncRNAs for which the predicted transcription start site is found within 1kb of an Encode-annotated enhancer. LncRNAs are divided into loci with predicted 1-to-1 orthologues in the rat (dark blue) and mouse-specific lncRNAs (light blue).

B. Same as A, for the percentage of multi-exonic loci, for mouse and rat.

C. Same as A, for the percentage of loci that have predicted bidirectional promoters, for mouse and rat.

D. Distribution of the raw expression divergence for protein-coding genes that are transcribed from the same bidirectional promoters as lncRNAs with 1-to-1 orthologues in mouse and rat (black), or as candidate species-specific lncRNAs (red).

E. Same as D, after correcting the expression divergence for the average expression level.

Supplementary Table List

Supplementary Table 1. List of RNA-seq samples generated specifically for this project, and used for all downstream expression analyses.

Supplementary Table 2. List of additional, previously published RNA-seq samples, included in the lncRNA detection pipeline.

Supplementary Table 3. Cell-type markers for the four organs analyzed here, derived from single-cell transcriptomics analyses.

Supplementary Table 4. List of putative organ/developmental stage markers. This list contains protein-coding genes with narrow expression distribution (organ/stage specificity value >= 0.85), which reach their maximum expression in the same organ and stage for mouse and rat.

Supplementary Table 5. Numbers of protein-coding genes and lncRNAs that have an average TPM expression level of at least 1 in each organ / developmental stage combination, for each species.

Supplementary Table 6. Sequence conservation scores (average PhastCons scores), for exons, introns, promoters and splice sites, for mouse protein-coding genes and lncRNAs.

Supplementary Table 7. List of 30 lncRNAs that are predicted to be 1-to-1 orthologues in mouse, rat and chicken.

Supplementary Table 8. Transcription factor binding site enrichment for the K-means clusters of lncRNAs and protein-coding genes that are differentially expressed among developmental stages in both mouse and rat.

Supplementary Table 9. Expression pattern and sequence conservation scores for protein-coding genes and lncRNAs, for mouse and rat 1-to-1 orthologues.

Supplementary Dataset List

Supplementary Datasets can be downloaded at the following address:

ftp://pbil.univ-lyon1.fr/pub/datasets/Darbellay_LncEvoDevo/supplementary_datasets

README files are provided with each dataset.

Supplementary Dataset 1. Complete gene annotations for mouse, rat and chicken.

Supplementary Dataset 2. Gene expression levels (raw and normalized TPM values, unique read counts).

Supplementary Dataset 3. Expression patterns (average across replicates, samples with maximum expression), expression specificity indexes, lists of organ/developmental stage markers, gene ontology enrichment for organ/developmental stage markers.

Supplementary Dataset 4. Results of the differential expression analyses across all developmental stages, or between consecutive developmental stages, for each organ and each species.

Supplementary Dataset 5. Predicted orthologous gene families and sequence conservation statistics, including PhastCons scores for all analyzed genomic regions.

Supplementary Dataset 6. Raw and normalized expression values (TPM) for orthologous protein-coding and lncRNA families.

Supplementary Dataset 7. Expression pattern divergence for mouse and rat orthologous genes.

Supplementary Dataset 8. Lists of candidate species-specific lncRNAs.

References

Benjamini Y, Hochberg Y. 1995. Controlling the false discovery rate: a practical and powerful approach to multiple testing. *J Roy Stat Soc B* **57**: 289+300.

Blanchette M, Kent WJ, Riemer C, Elnitski L, Smit AFA, Roskin KM, Baertsch R, Rosenbloom K, Clawson H, Green ED, et al. 2004. Aligning multiple genomic sequences with the threaded blockset aligner. *Genome Res* **14**: 708–715.

Casper J, Zweig AS, Villarreal C, Tyner C, Speir ML, Rosenbloom KR, Raney BJ, Lee CM, Lee BT, Karolchik D, et al. 2018. The UCSC Genome Browser database: 2018 update. *Nucleic Acids Res* **46**: D762–D769.

Dray S, Dufour AB. 2007. The ade4 package: implementing the duality diagram for ecologists. *J Stat Softw* **22**: 1–20.

Green CD, Ma Q, Manske GL, Shami AN, Zheng X, Marini S, Moritz L, Sultan C, Gurczynski SJ, Moore BB, et al. 2018. A comprehensive roadmap of murine spermatogenesis defined by single-cell RNA-Seq. *Dev Cell* **46**: 651-667.e10.

Hartigan J, Wong M. 1979. Algorithm AS 136: A K-Means Clustering Algorithm. *J R Stat Soc Ser C Appl Stat* **28**.

Heinz S, Benner C, Spann N, Bertolino E, Lin YC, Laslo P, Cheng JX, Murre C, Singh H, Glass CK. 2010. Simple combinations of lineage-determining transcription factors prime cis-regulatory elements required for macrophage and B cell identities. *Mol Cell* **38**: 576–589.

Liao B-Y, Scott NM, Zhang J. 2006. Impacts of gene essentiality, expression pattern, and gene compactness on the evolutionary rate of mammalian proteins. *Mol Biol Evol* **23**: 2072–2080.

R Core Team. 2018. *R: A Language and Environment for Statistical Computing*. https://www.R-project.org/.

Shen Y, Yue F, McCleary DF, Ye Z, Edsall L, Kuan S, Wagner U, Dixon J, Lee L, Lobanenkov VV, et al. 2012. A map of the cis-regulatory sequences in the mouse genome. *Nature* **488**: 116–120.

Siepel A, Bejerano G, Pedersen JS, Hinrichs AS, Hou M, Rosenbloom K, Clawson H, Spieth J, Hillier LW, Richards S, et al. 2005. Evolutionarily conserved elements in vertebrate, insect, worm, and yeast genomes. *Genome Res* **15**: 1034–50.

Smedley D, Haider S, Durinck S, Pandini L, Provero P, Allen J, Arnaiz O, Awedh MH, Baldock R, Barbiera G, et al. 2015. The BioMart community portal: an innovative alternative to large, centralized data repositories. *Nucleic Acids Res* **43**: W589-598.

Tabula Muris Consortium. 2018. Single-cell transcriptomics of 20 mouse organs creates a Tabula Muris. *Nature* **562**: 367–372.

Supplementary Figures

Darbellay and Necsulea, Supplementary Figure 1

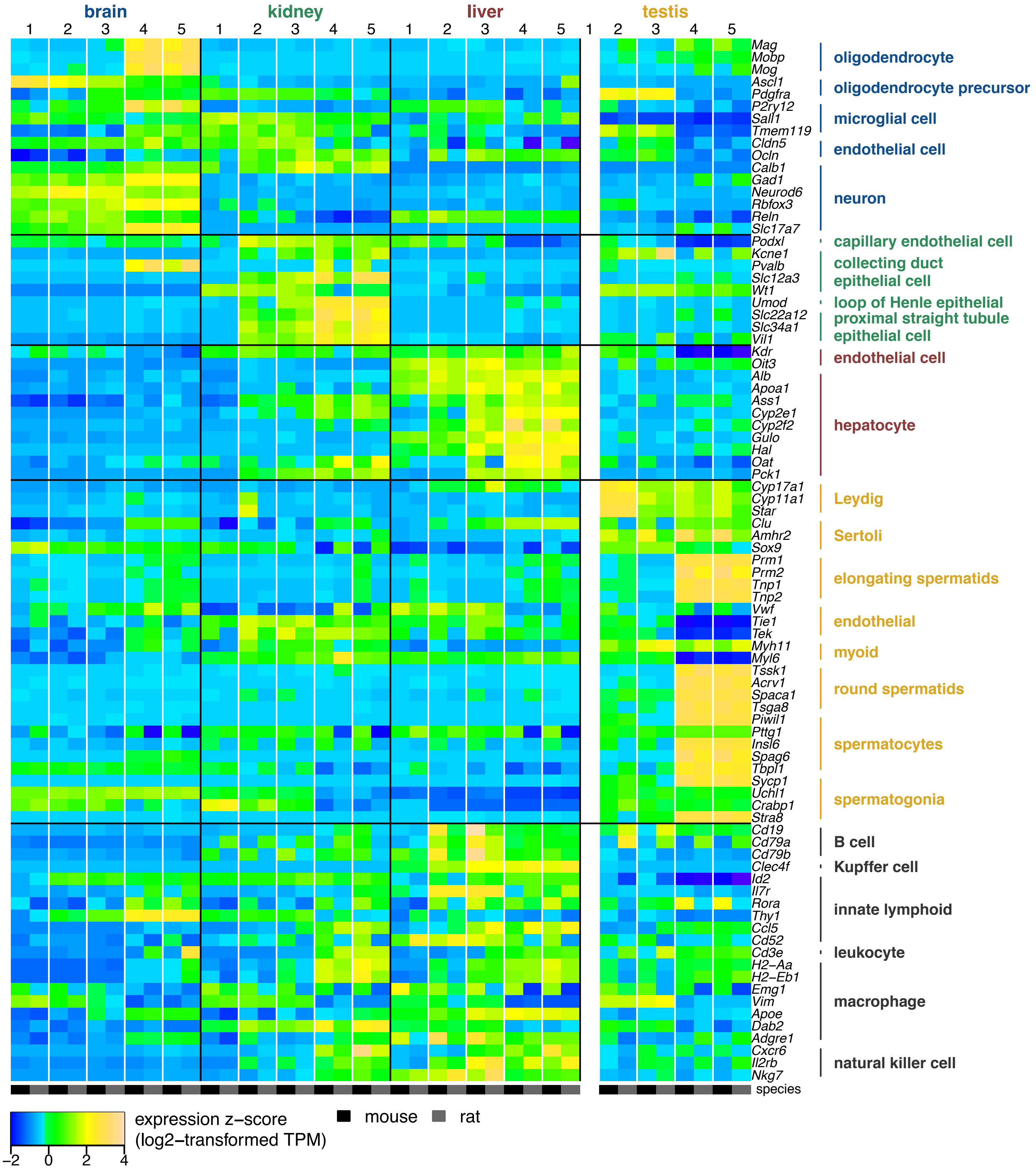

Darbellay and Necsulea, Supplementary Figure 2

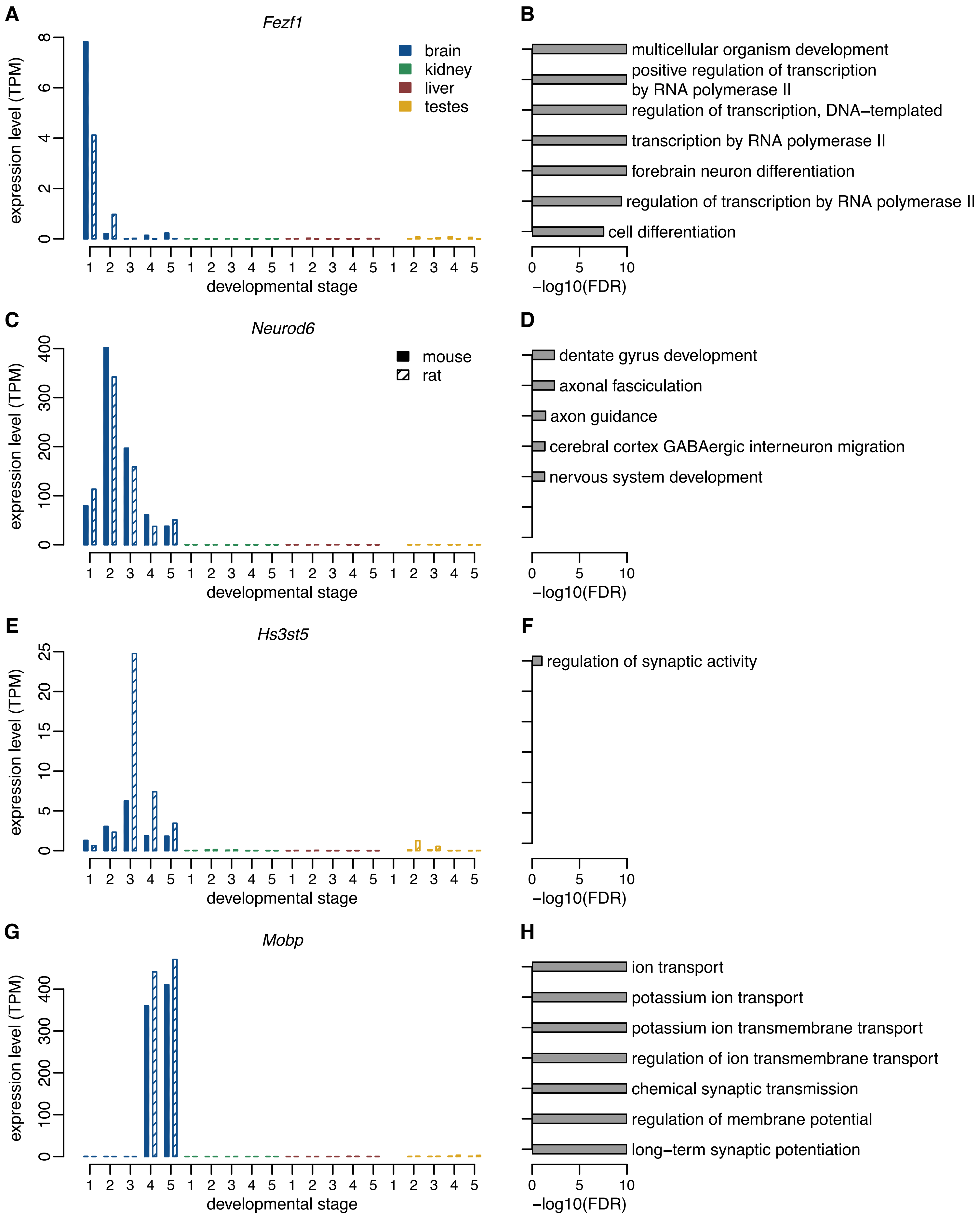

Darbellay and Necsulea, Supplementary Figure 3

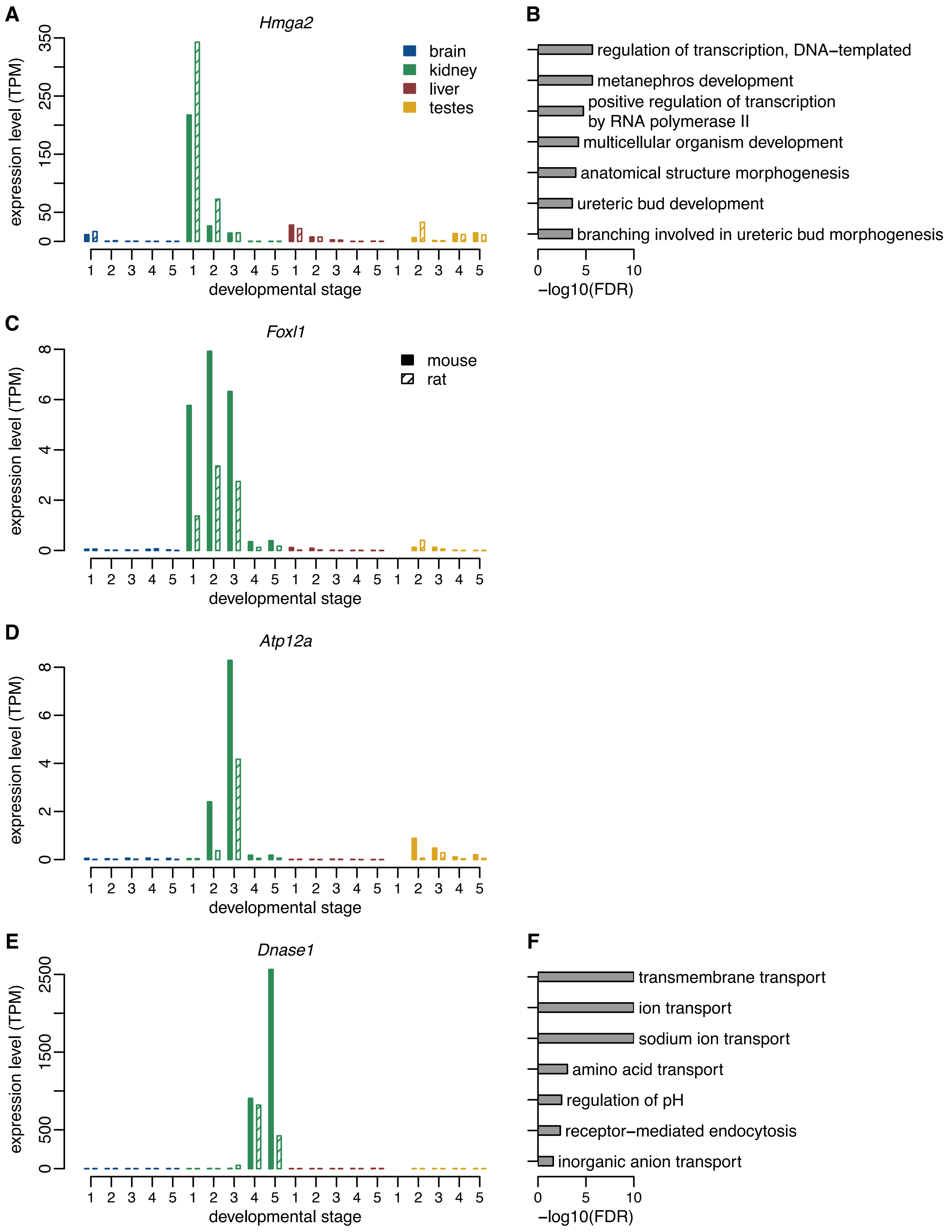

Darbellay and Necsulea, Supplementary Figure 4

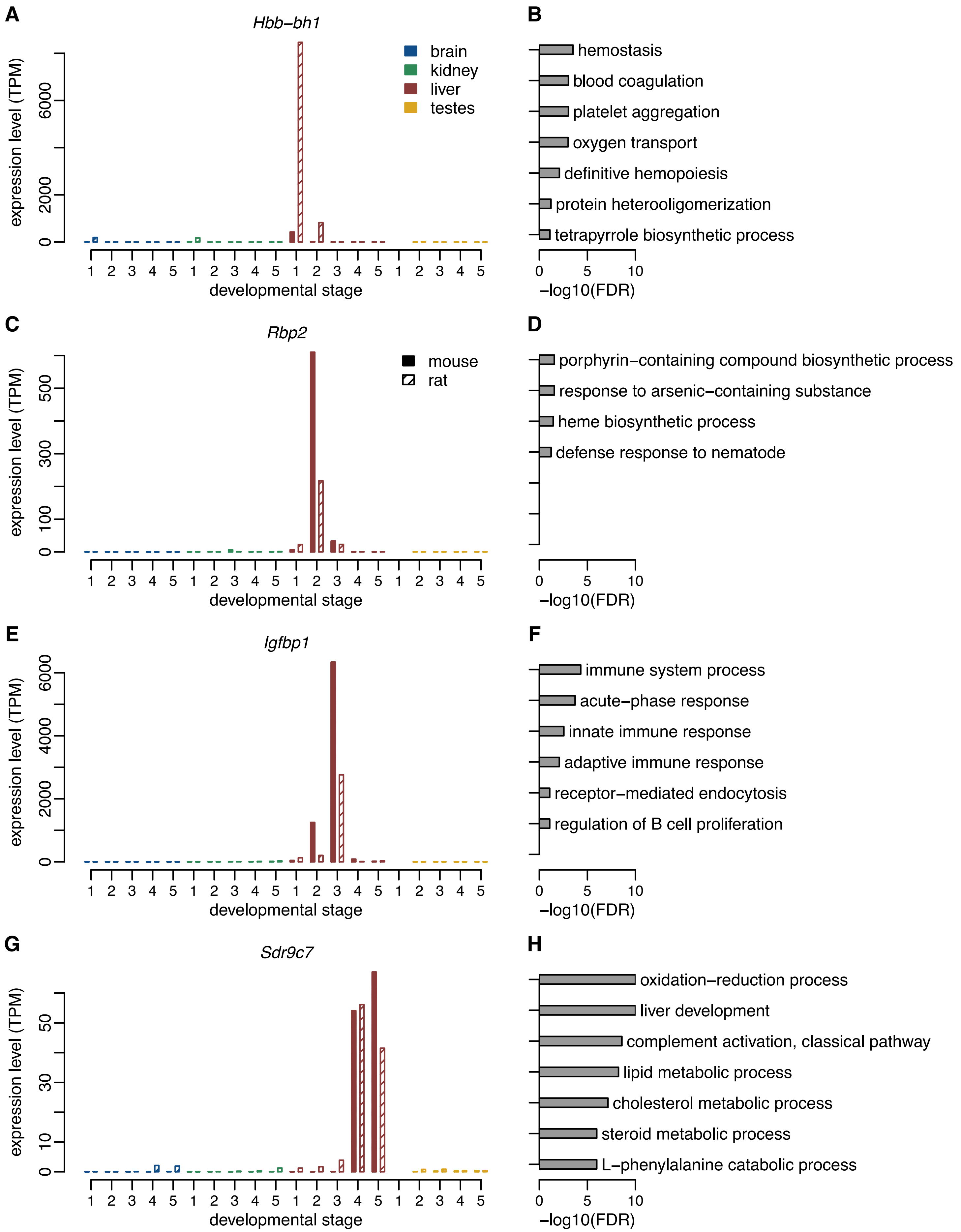

Darbellay and Necsulea, Supplementary Figure 5

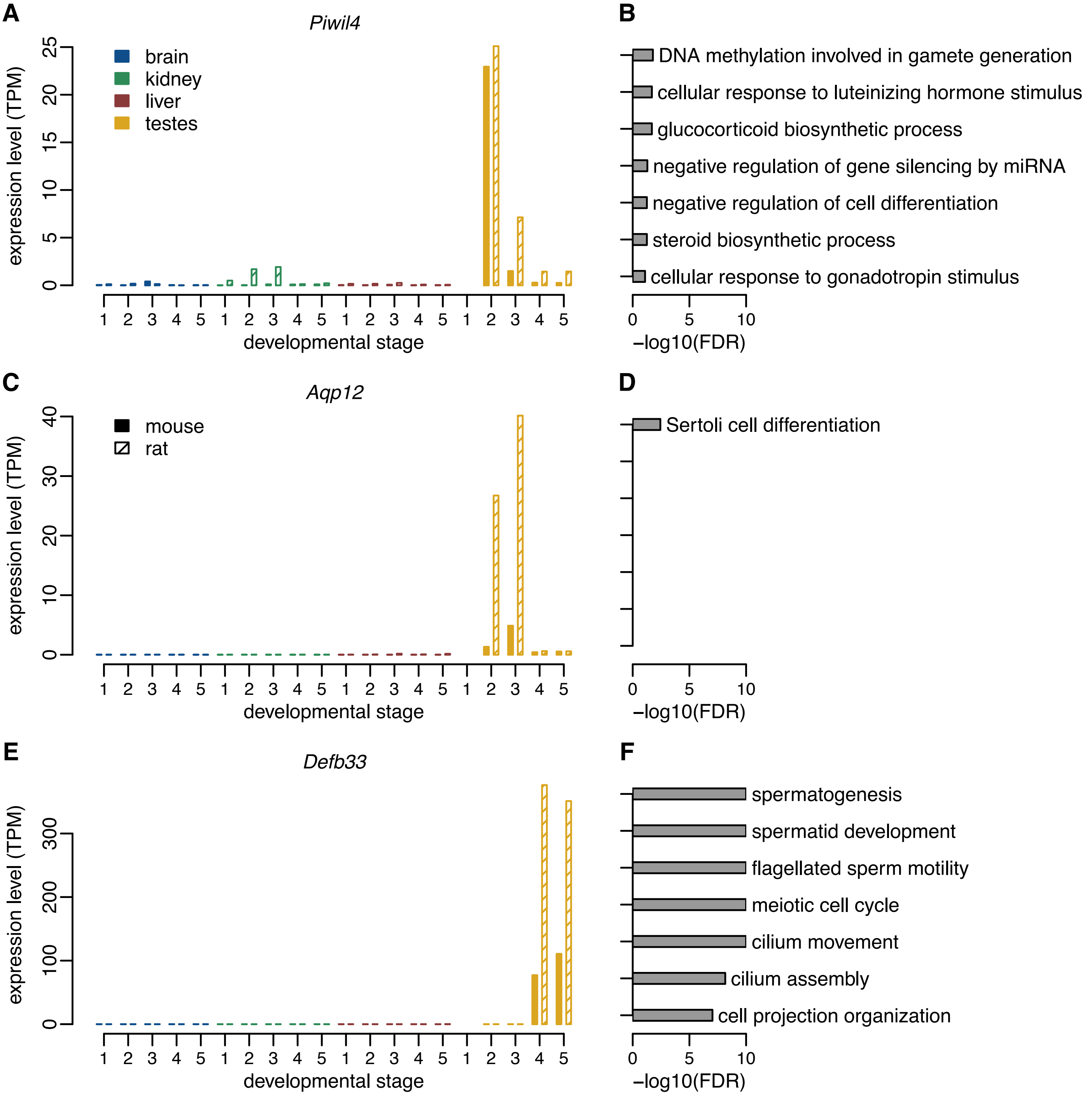

Darbellay and Necsulea, Supplementary Figure 6

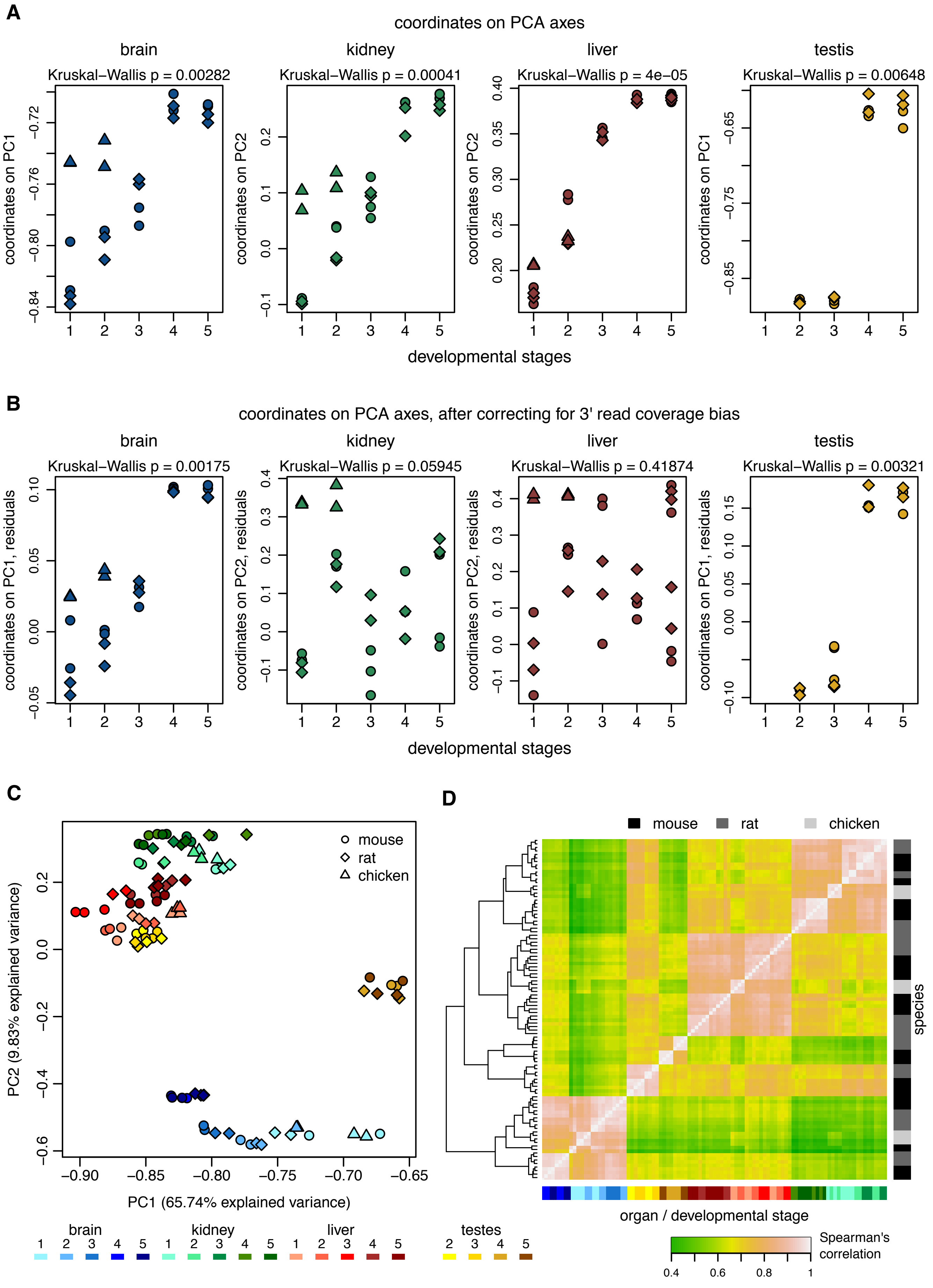

Darbellay and Necsulea, Supplementary Figure 7

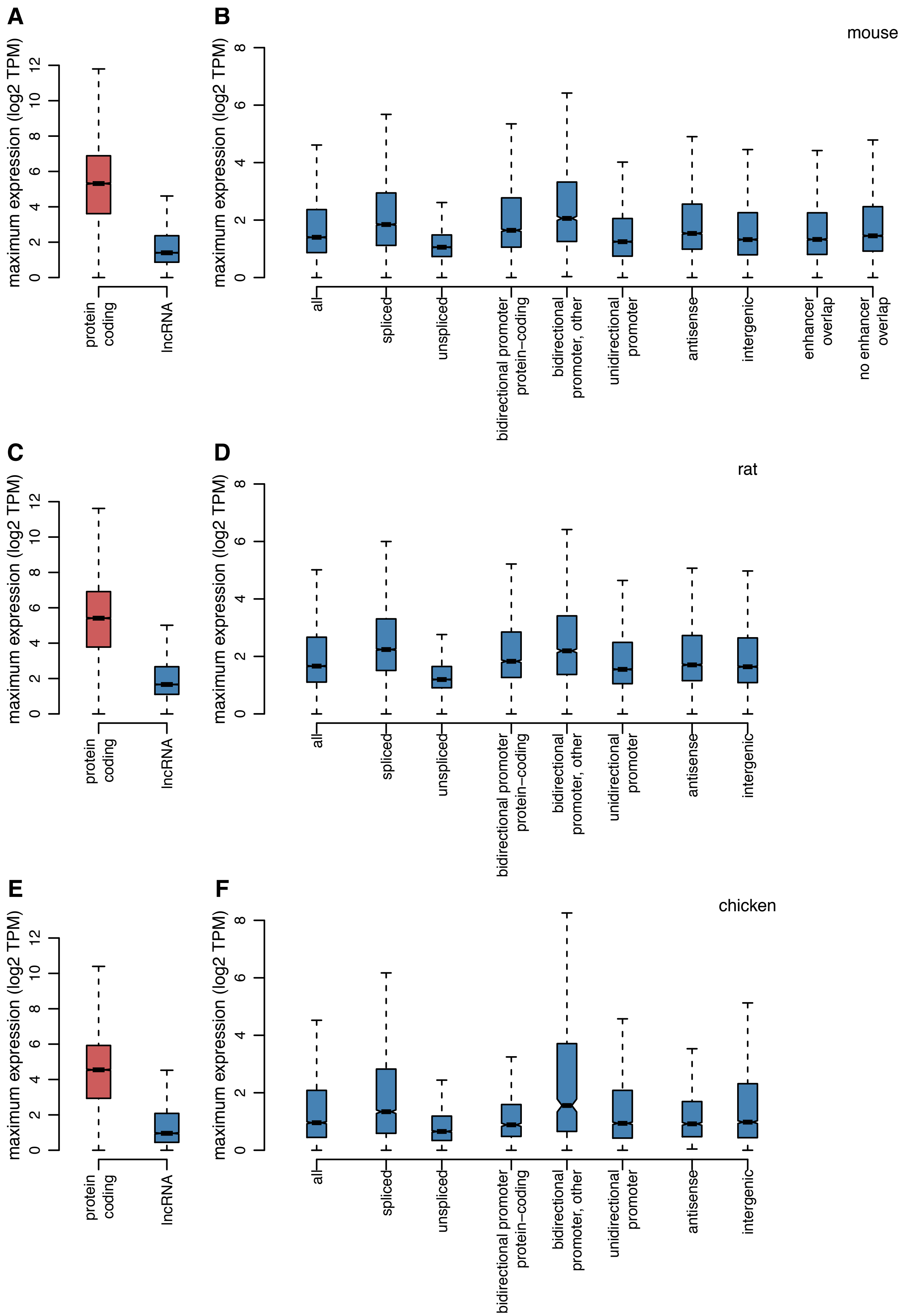

Darbellay and Necsulea, Supplementary Figure 8

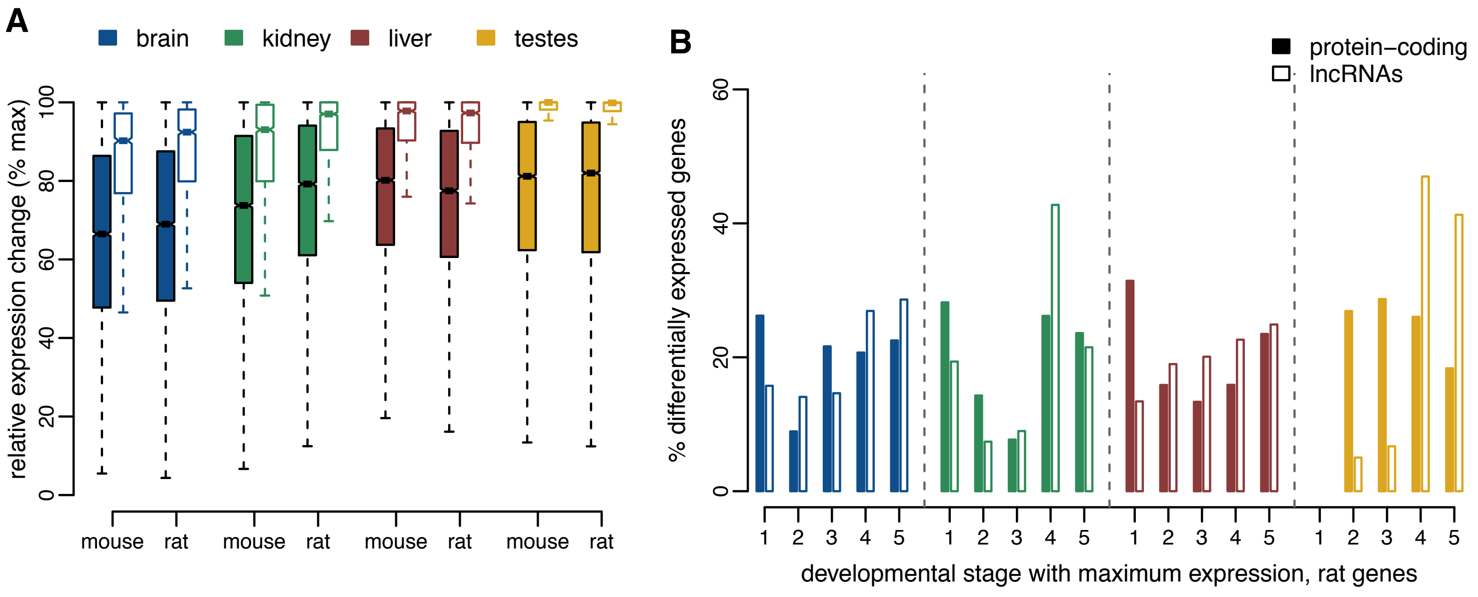

Darbellay and Necsulea, Supplementary Figure 9

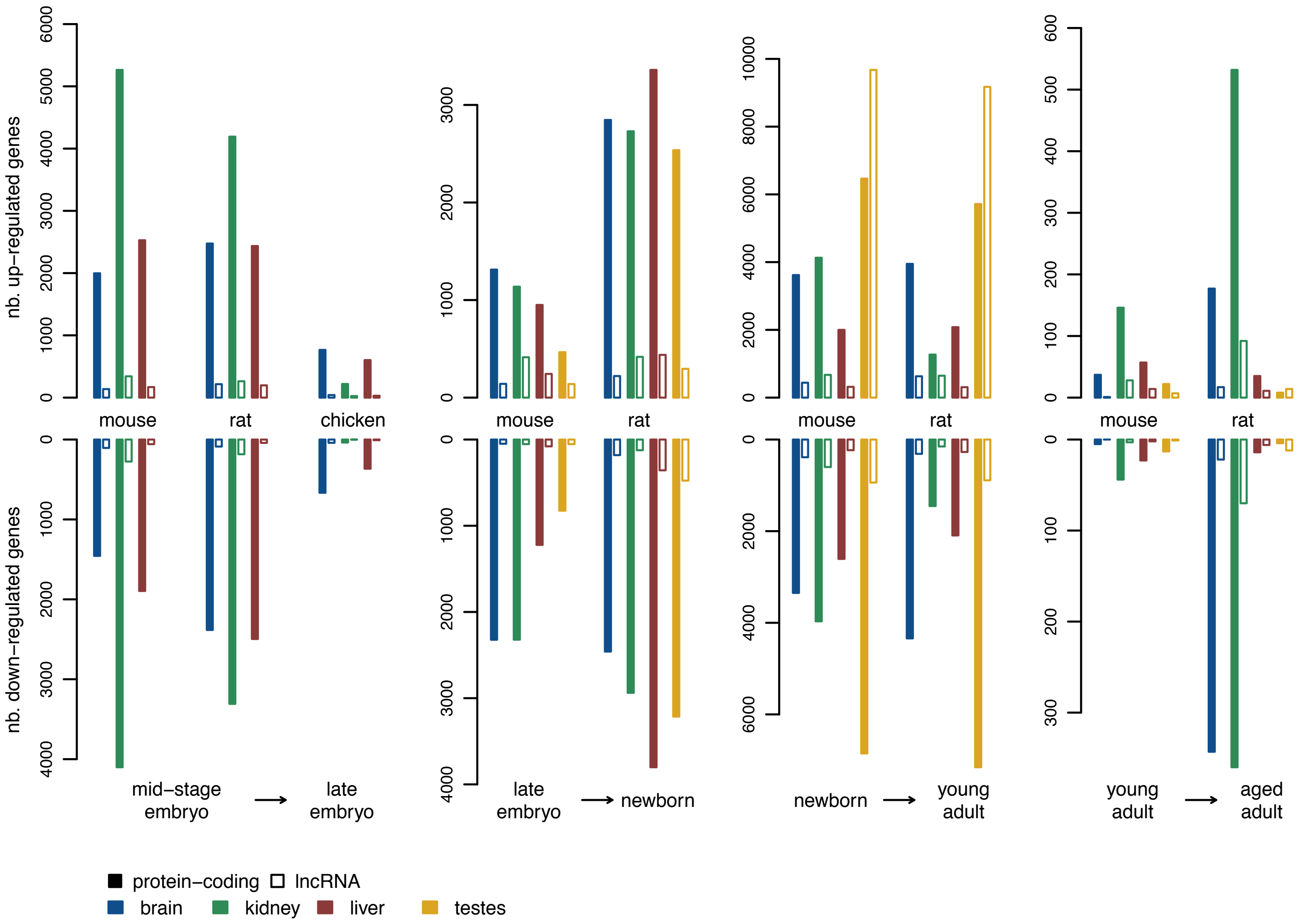

Darbellay and Necsulea, Supplementary Figure 10

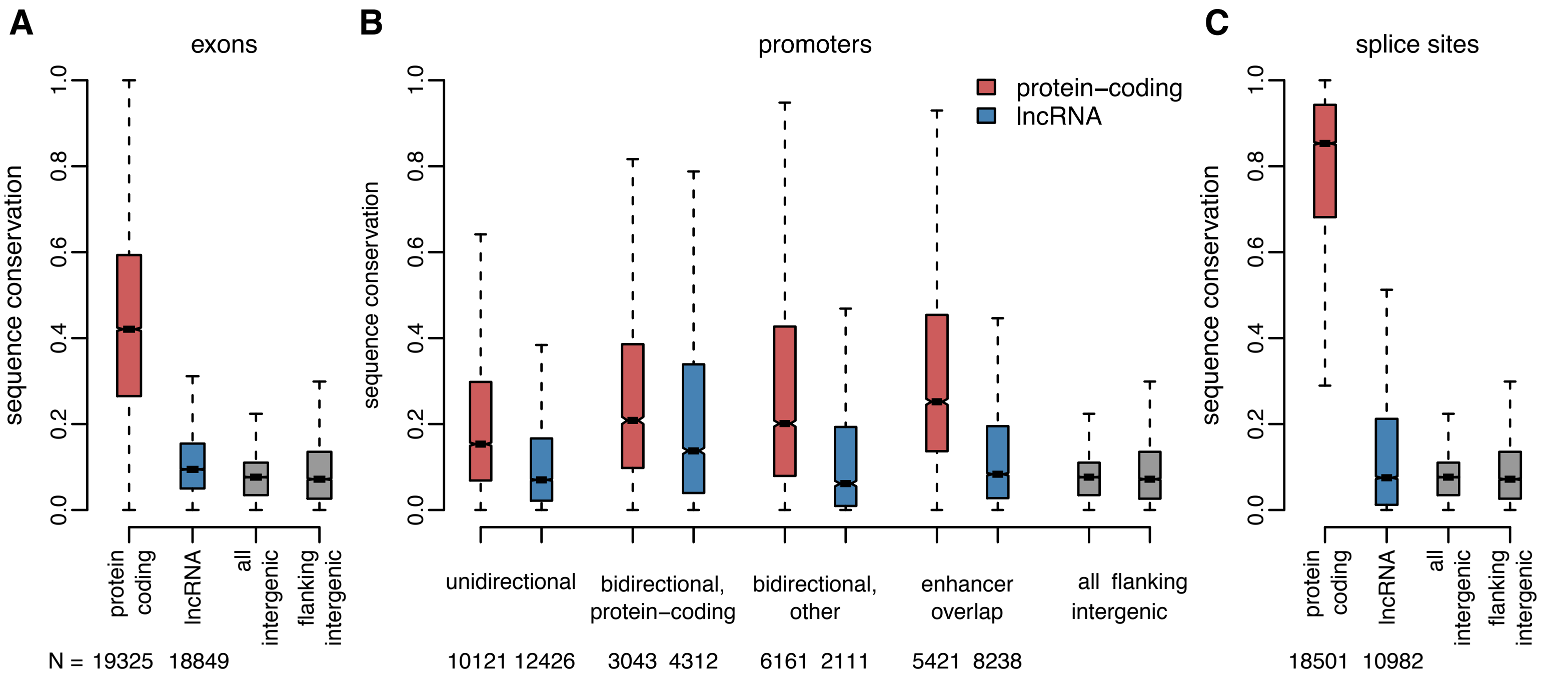

Darbellay and Necsulea, Supplementary Figure 11

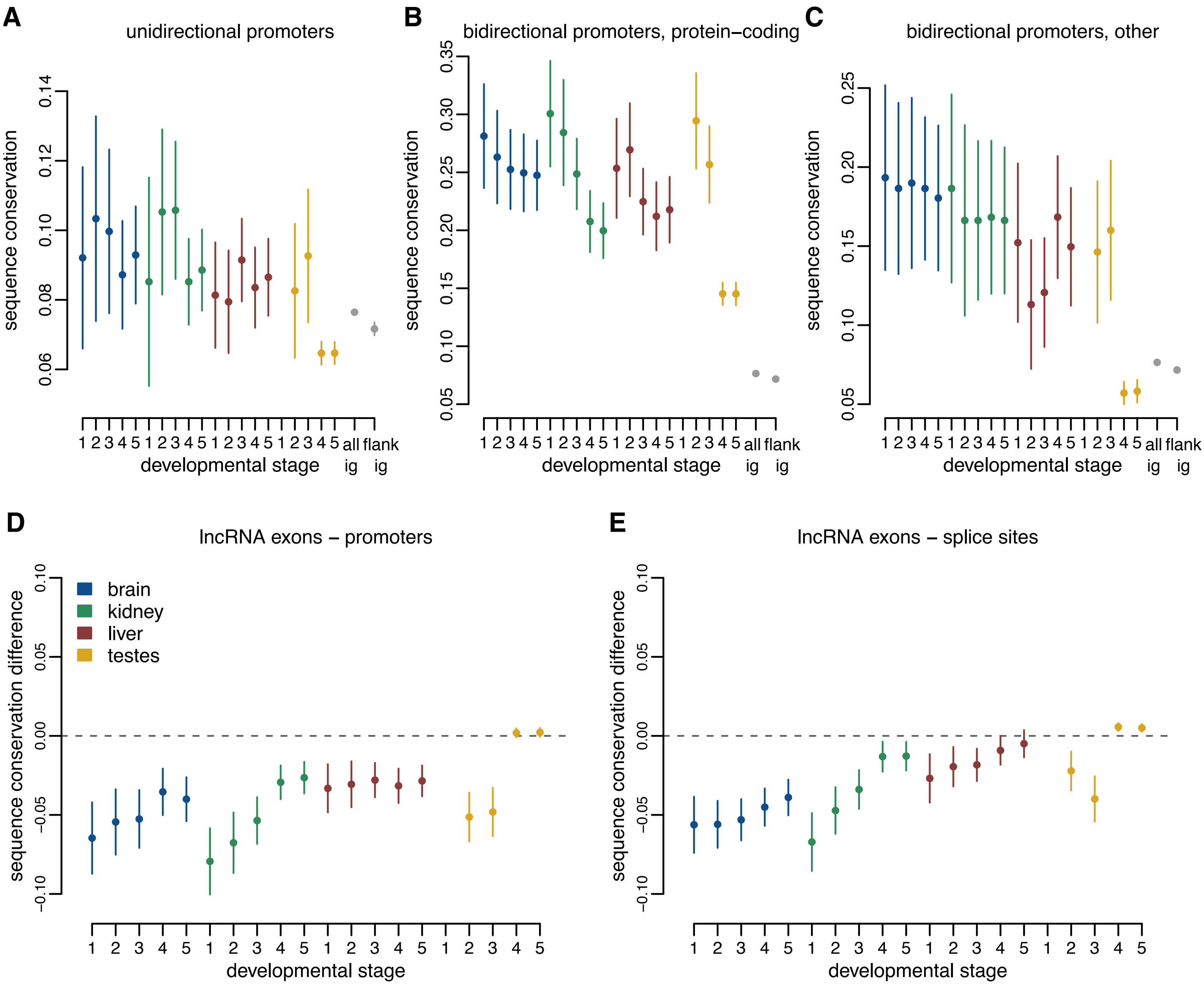

Darbellay and Necsulea, Supplementary Figure 12

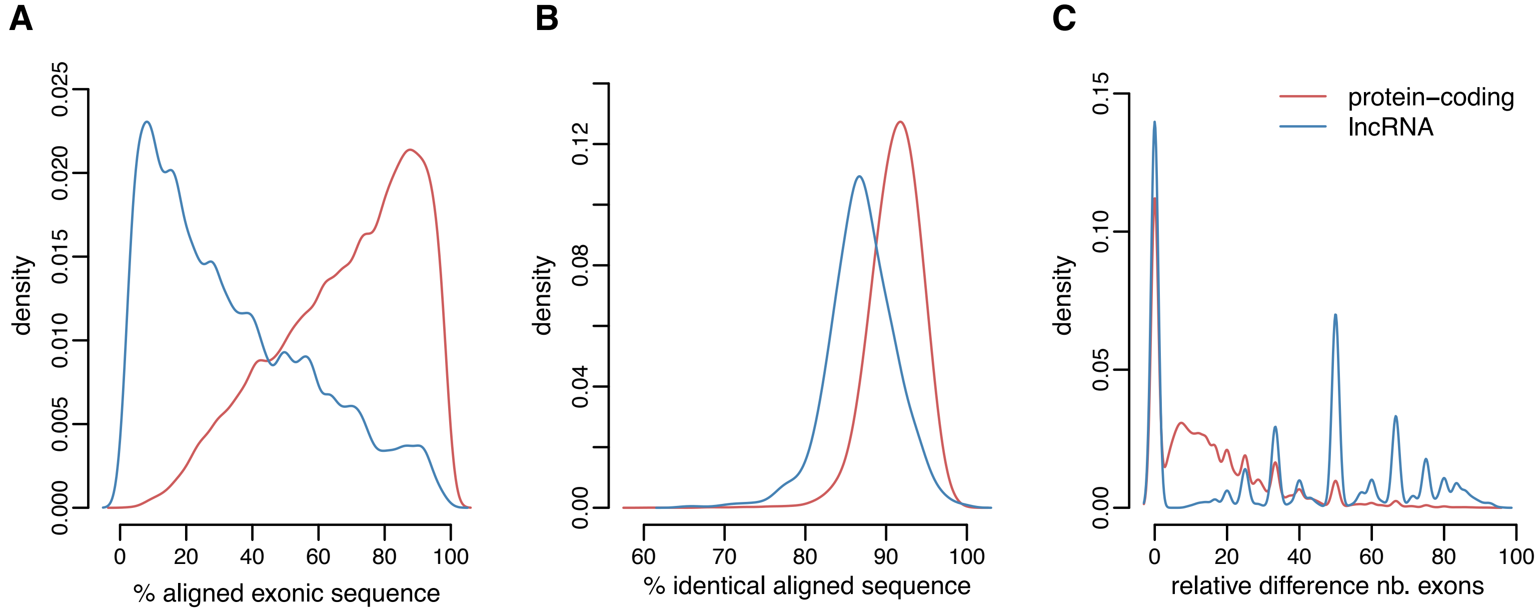

Darbellay and Necsulea, Supplementary Figure 13

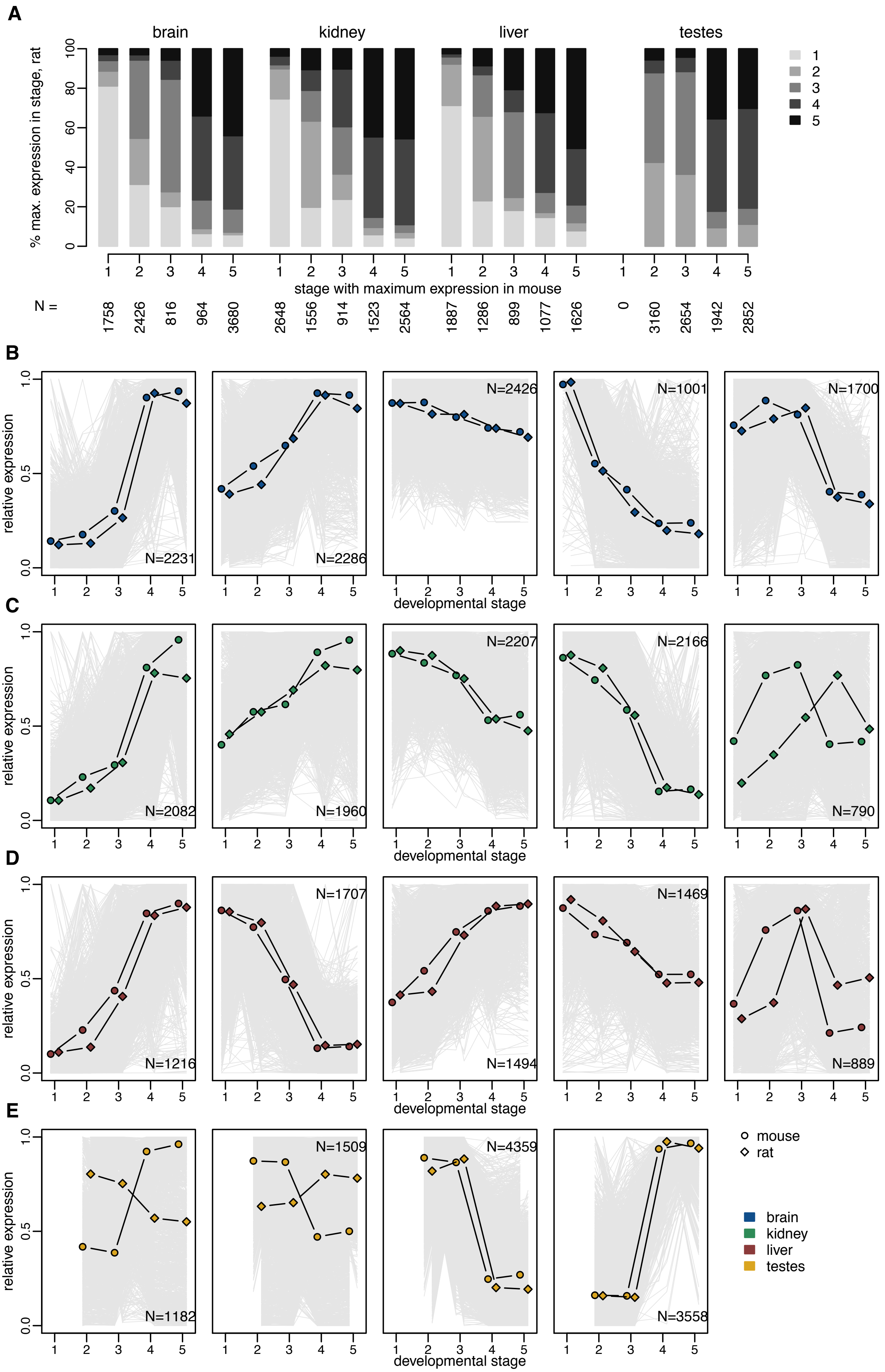

Darbellay and Necsulea, Supplementary Figure 14

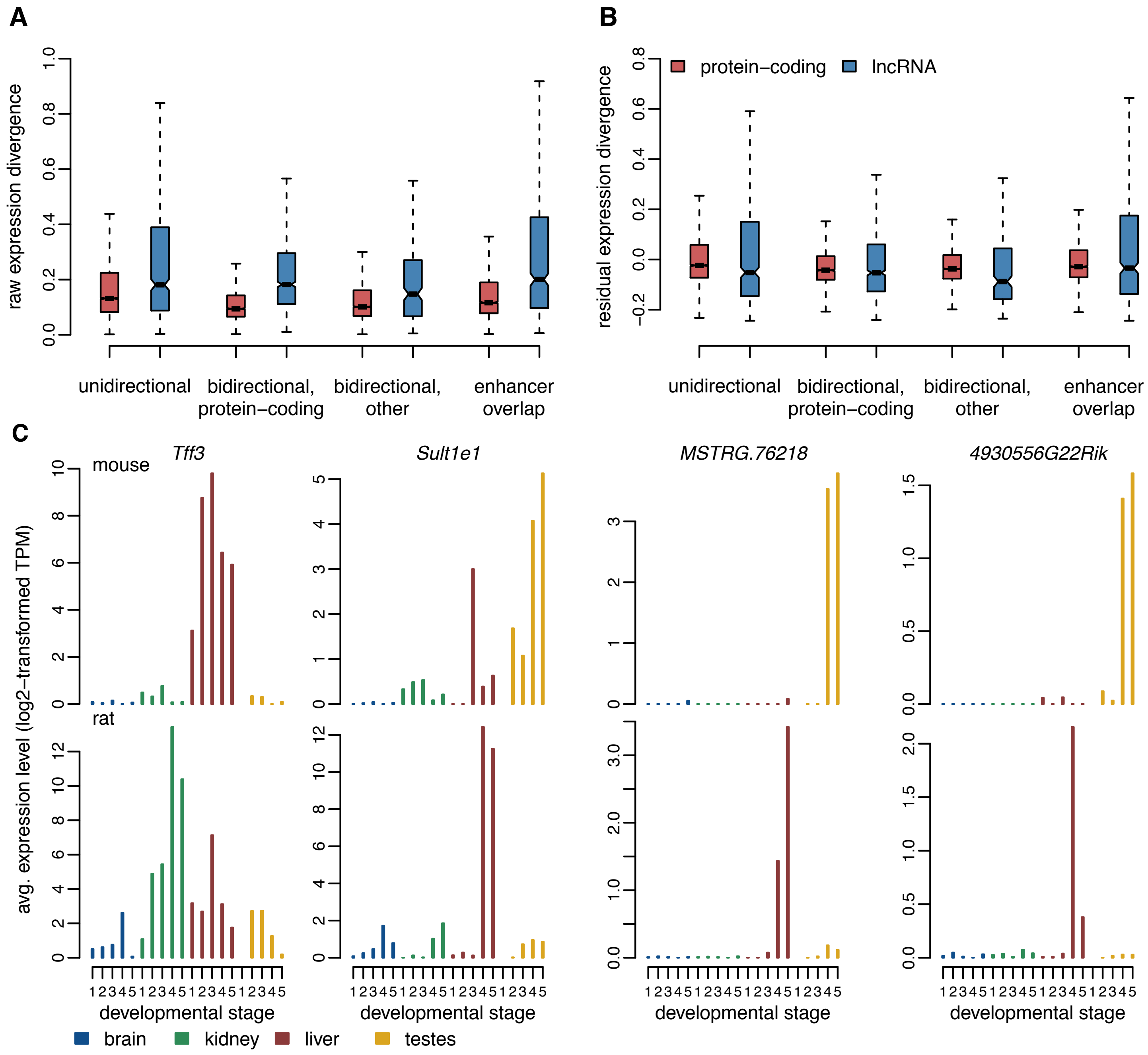

Darbellay and Necsulea, Supplementary Figure 15

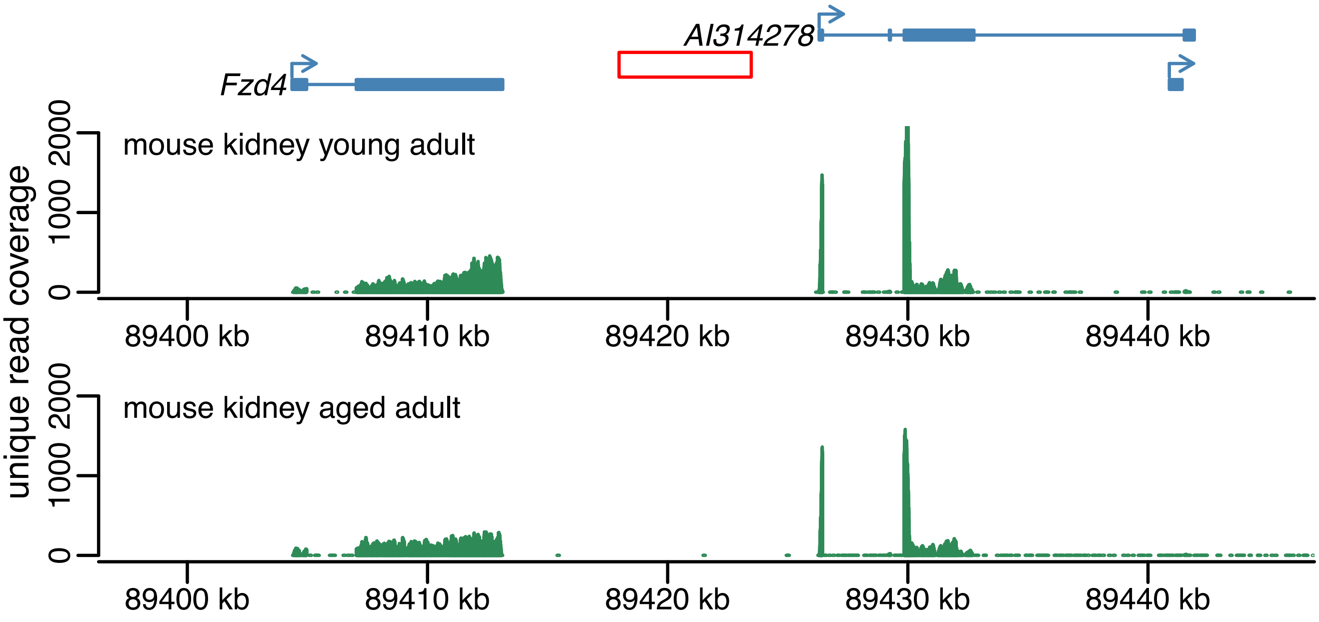

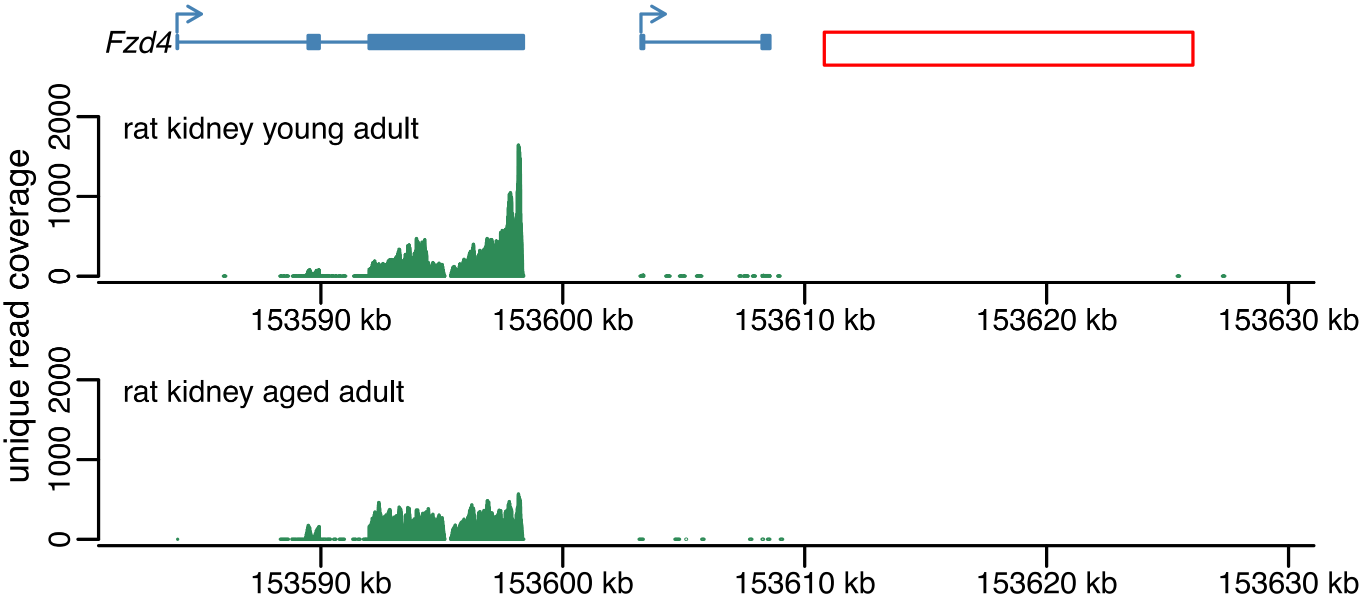

Darbellay and Necsulea, Supplementary Figure 16

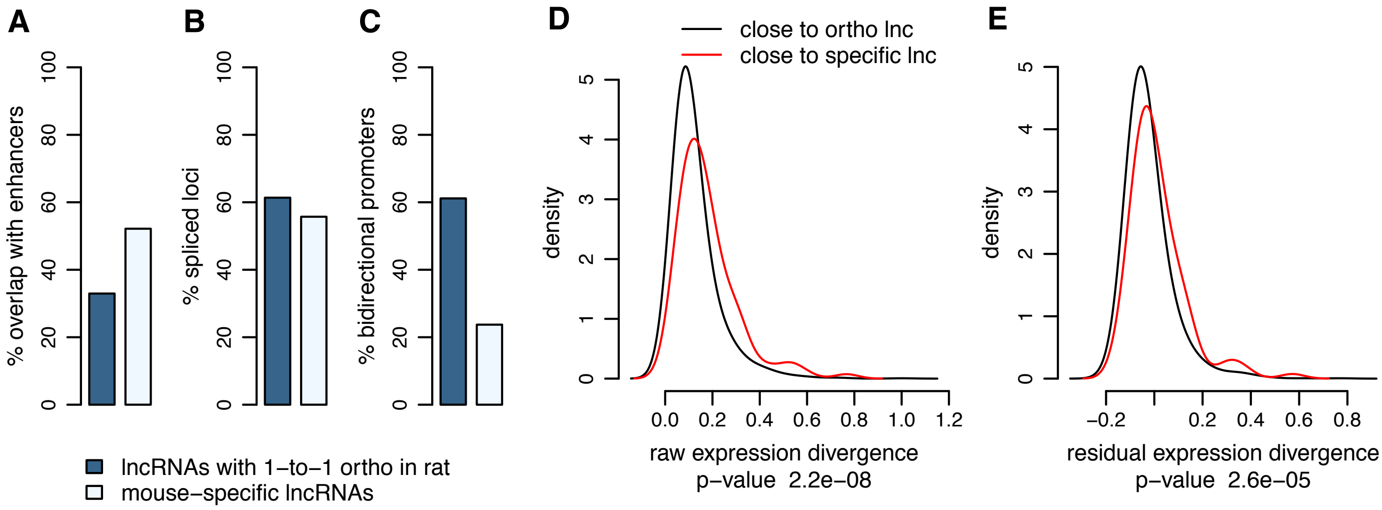

Supplement: msz212_Supplementary_Data [file msz212_supplementary_data.zip › msz212-Suppl_data/SupplementaryMaterial.pdf]
